# Obstructive nephropathy and molecular pathophysiology of renal interstitial fibrosis

**DOI:** 10.1152/physrev.00027.2022

**Published:** 2023-07-13

**Authors:** Rikke Nørregaard, Henricus A. M. Mutsaers, Jørgen Frøkiær, Tae-Hwan Kwon

**Affiliations:** ^1^Department of Clinical Medicine, Aarhus University, Aarhus, Denmark; ^2^Department of Renal Medicine, Aarhus University Hospital, Aarhus, Denmark; ^3^Department of Biochemistry and Cell Biology, School of Medicine, Kyungpook National University, Taegu, Korea

**Keywords:** chronic kidney disease, fibrosis, hemodynamics, homeostasis, ureteral obstruction

## Abstract

The kidneys play a key role in maintaining total body homeostasis. The complexity of this task is reflected in the unique architecture of the organ. Ureteral obstruction greatly affects renal physiology by altering hemodynamics, changing glomerular filtration and renal metabolism, and inducing architectural malformations of the kidney parenchyma, most importantly renal fibrosis. Persisting pathological changes lead to chronic kidney disease, which currently affects ∼10% of the global population and is one of the major causes of death worldwide. Studies on the consequences of ureteral obstruction date back to the 1800s. Even today, experimental unilateral ureteral obstruction (UUO) remains the standard model for tubulointerstitial fibrosis. However, the model has certain limitations when it comes to studying tubular injury and repair, as well as a limited potential for human translation. Nevertheless, ureteral obstruction has provided the scientific community with a wealth of knowledge on renal (patho)physiology. With the introduction of advanced omics techniques, the classical UUO model has remained relevant to this day and has been instrumental in understanding renal fibrosis at the molecular, genomic, and cellular levels. This review details key concepts and recent advances in the understanding of obstructive nephropathy, highlighting the pathophysiological hallmarks responsible for the functional and architectural changes induced by ureteral obstruction, with a special emphasis on renal fibrosis.

## Graphical abstract

**Figure:**
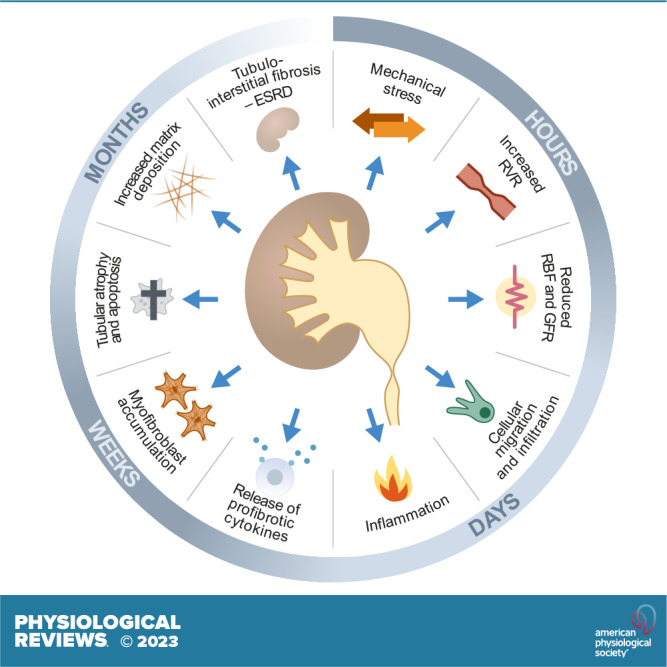

CLINICAL HIGHLIGHTS

- Chronic kidney disease (CKD) affects 10% of the world population and is a leading cause of death. CKD, characterized by inflammation and renal fibrosis, can progress to end-stage renal disease (ESRD), which is fatal if left untreated.
- Obstructive nephropathy, the renal disease caused by impaired urine flow, can become manifest at all ages, and the causes, either benign or malignant, are manifold.
- Kidney function and architecture are severely impacted by ureteral obstruction; however, the extent of the alterations, and thus the pathological consequences, depend greatly on the duration, degree, and site of the obstruction.
- Experimental ureteral obstruction has been used as a model to study renal pathophysiology since the 1800s and is currently mainly used to elucidate the molecular and cellular mechanisms underlying renal fibrosis as well as for identifying therapeutic targets. At the time of writing, almost 18,500 publications on experimental ureteral obstruction are indexed in PubMed, testifying to the widespread use of this model. However, the often-used complete and irreversible model of ureteral obstruction has limitations when it comes to studying tubular injury and repair.
- Several cell types and signaling pathways are involved in renal fibrosis, which greatly hampers the development of efficacious therapeutics. Combining models of obstructive nephropathy with next-generation analysis techniques will greatly advance our understanding of renal fibrosis and support drug development.

## 1. INTRODUCTION

The global incidence of chronic kidney disease (CKD) is steadily increasing, presenting a significant clinical challenge because CKD may lead to end-stage renal disease (ESRD), which requires interventions such as dialysis and kidney transplantation to prevent death. CKD affects more than 0.5 billion people and has a global prevalence rate approaching 10% (1, 2). A prominent feature of CKD is inflammation of the tubulointerstitial compartment, which leads to renal fibrosis, a complex process involving the loss of capillary networks, decline in renal functions, and progressive accumulation of fibrillary collagens, activated myofibroblasts, and inflammatory cells (3, 4). As fibrosis progresses, kidney functions gradually deteriorate, characterized by reduced renal blood flow (RBF) and tissue perfusion, decreased glomerular filtration rate (GFR), impaired tubular handling of water and electrolytes, and elevated urinary protein excretion.

**Figure 1.**
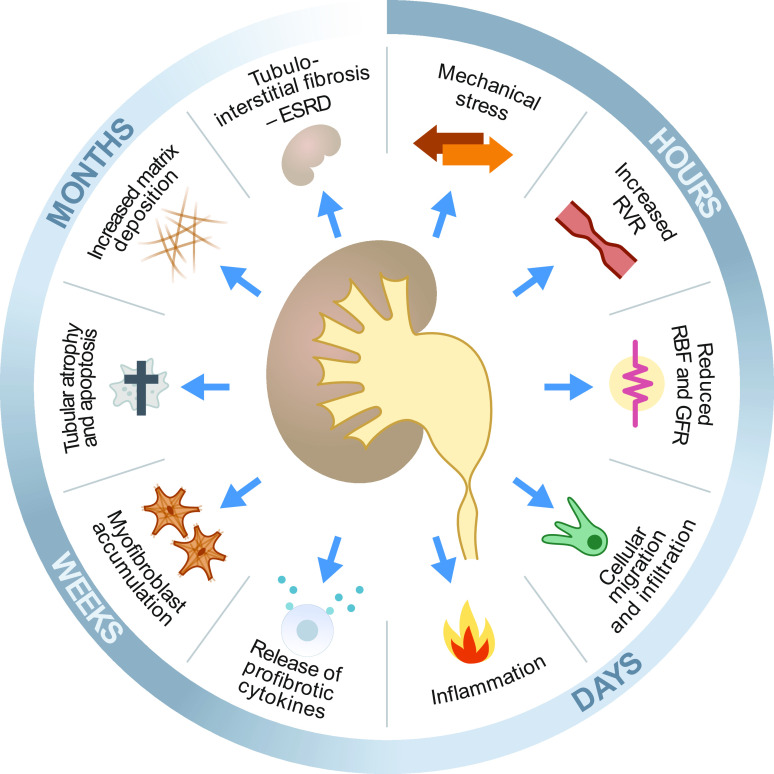
Hallmarks of obstructive nephropathy showing key characteristics of functional and cellular consequences of ureteral obstruction as a time lapse from hours to months. ESRD, end-stage renal disease; GFR, glomerular filtration rate; RBF, renal blood flow; RVR, renal vascular resistance.

There are numerous diseases causing CKD, including hypertension, diabetes, and cardiovascular disease, and the speed and severity of CKD development vary among these different diseases. Another important condition causing CKD is obstructive nephropathy, which is also characterized by renal dysfunction and renal interstitial fibrosis. Obstructive nephropathy is caused by chronic urinary tract obstruction that is the consequence of numerous urinary tract diseases, including congenital anatomic abnormalities, renal calculi, prostatic hyperplasia, and bladder tumors, and it is characterized by a complex array of pathophysiological processes leading to ESRD (FIGURE 1). The understanding of these functional changes has been significantly enhanced through advances in cell and molecular biology, and much of this knowledge was accumulated through animal studies.

Unilateral ureteral obstruction (UUO), also a model for experimental hydronephrosis, is among the most important animal models for identifying novel mechanisms underlying renal fibrosis. The use of different species with UUO as an experimental model to examine renal (patho)physiology dates back centuries. In 1919, Dr. Hinman wrote in the *Journal of Urology* “The literature relative to experimental hydronephrosis is enormous. Original contributions personally reviewed number more than three hundred which is only a partial list” (5). In the same paper, the earliest work referred to is from 1859. The literature concerning ureteral obstruction (UO) has expanded dramatically ever since, with almost 18,500 publications indexed in PubMed at the time of writing this review. The fact that the UUO model has been in use for such a long time underscores its value in studying the molecular mechanisms of renal fibrosis, but it is important to acknowledge that the model has limitations when it comes to investigating tubular injury and repair.

**Figure 2.**
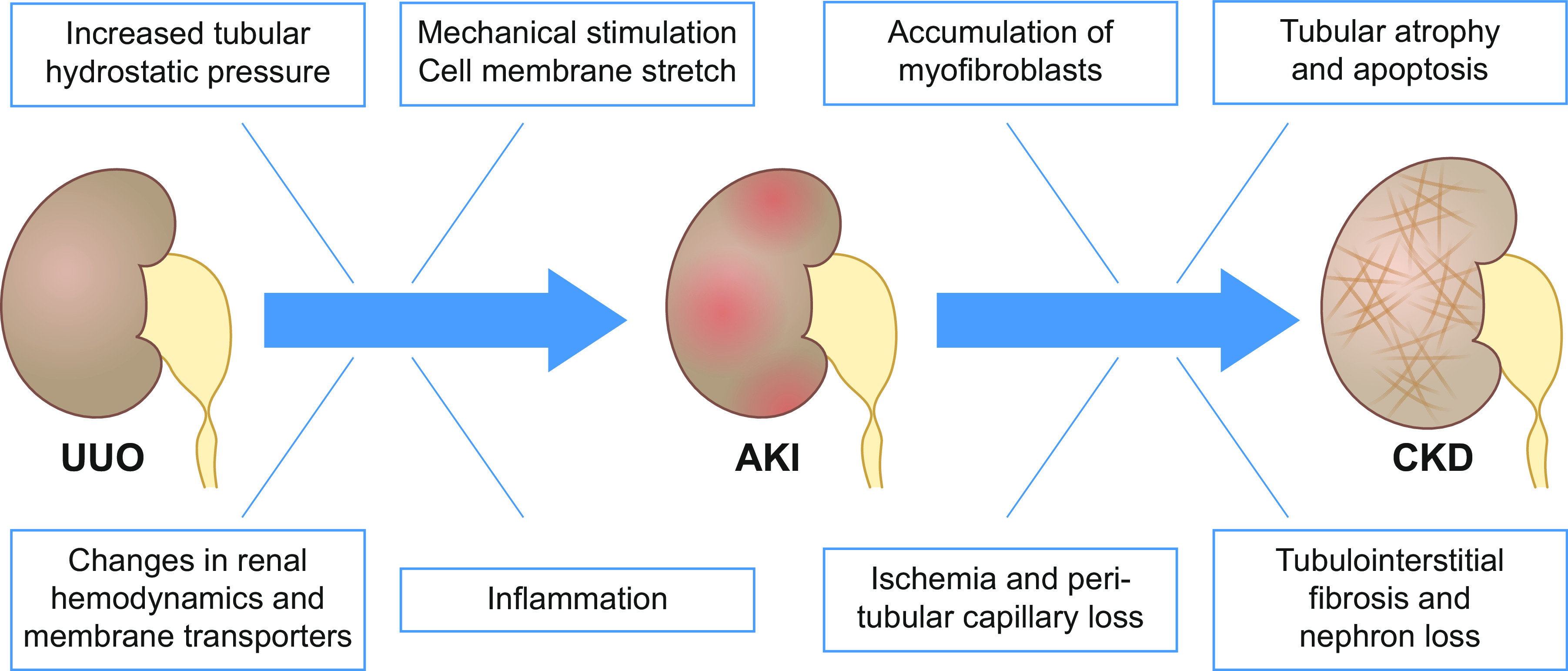
Schematic overview of the functional, cellular, and molecular consequences of unilateral ureteral obstruction (UUO) leading to acute kidney injury (AKI) or chronic kidney disease (CKD).

This review provides an overview of the hallmarks of obstructive nephropathy, highlighting the key molecular mechanisms currently known to contribute to the decline in kidney function and the progression of renal interstitial fibrosis (FIGURE 2).

## 2. CLINICAL OBSTRUCTIVE NEPHROPATHY

Obstructive uropathy refers to the presence of structural or functional changes in the urinary tract that interrupt the normal flow of urine (6), whereas obstructive nephropathy is the renal disease caused by impaired urine flow. Obstructive nephropathy can manifest at all ages, stretching from the antenatal period to adulthood, and is caused by chronic urinary tract obstruction, which may be associated with hydronephrosis (i.e., dilation of the urinary tract) (6). Obstructive nephropathy has many causes, both benign and malignant. The most common causes of intrinsic obstruction in children are congenital anatomic abnormalities, whereas renal calculi (6), affecting ∼10% of adults worldwide (7), are the most common cause in young adults. In older adults, benign prostatic hyperplasia, prostate cancer, and bladder tumors are common causes of intrinsic obstruction (6). On the other hand, extrinsic obstruction is caused by processes outside the urinary tract, such as retroperitoneal fibrosis and Crohn’s disease (8–10), as well as pregnancy (11). Moreover, obstructive uropathy can be classified according to the underlying cause (congenital or acquired), duration (acute or chronic), degree (partial or complete), and site (unilateral or bilateral). Clinical signs and symptoms may be absent or vary from flank pain, urinary retention, overflow incontinence, and decreased urine flow to complicated urinary tract infections and acute renal failure. The overall incidence and prevalence of obstructive nephropathy are difficult to assess accurately. A review based on 59,064 autopsies, ranging from neonates to elderly, identified hydronephrosis in 3.1% (3.3% in males and 2.9% in females) (12). Provided that hydronephrosis can be used as a surrogate marker of obstruction, the study showed that until the age of 20 yr there was no substantial sex difference in the frequency of abnormalities. Between the ages of 20 and 60 yr urinary tract obstruction was more frequent among women than among men, and above the age of 60 yr prostatic disease raised the frequency of urinary tract obstruction among men above that observed among women. Because a high proportion of these autopsy-detected cases of obstruction likely went undetected during life, the overall prevalence of urinary tract obstruction is most likely far greater than reports suggest. This conclusion is reinforced by the fact that obstruction can be asymptomatic and transient, for instance, when the obstruction is caused by pregnancy or renal calculi. To date, surgery remains the primary intervention for urinary tract obstruction; however, for many patients with moderate or severe loss of renal function, improving ureteric drainage does not necessarily lead to an improvement in renal function (13). Thus, there remains a clinical need for improved treatment modalities. One potential drug candidate that holds promise for renal function recovery following relief of obstruction is losartan, an angiotensin II (ANG II) receptor blocker with proven efficacy in both experimental UUO (14–16) (further details regarding the model can be found below) and patients with UO (17). Patients with UO due to benign prostatic hyperplasia often receive α-blocker therapy to improve urinary flow. Interestingly, recent findings have revealed that pharmacological blockade of α1-adrenergic receptors can reduce UUO-induced renal fibrosis in mice (18). Furthermore, patients experiencing both UO and renal colic, frequently resulting from kidney stones, are commonly prescribed analgesics such as nonsteroidal anti-inflammatory drugs (NSAIDs) (19). NSAIDs function by modulating the prostaglandin system. However, it is crucial to acknowledge that these drugs can also potentially induce acute kidney injury (AKI) (20–22). Therefore, it is imperative to exercise caution when using this class of drugs. The duality of the prostaglandin system in renal disease is elaborated upon below in this review.

## 3. EXPERIMENTAL MODELS OF URETERAL OBSTRUCTION

### 3.1. Animal Models of Ureteral Obstruction

Even though the clinical features of UO have been recognized for ages, our understanding of the pathophysiology of renal dysfunction has been markedly enhanced by detailed studies of experimental animal models of UO. The effects of UO on renal anatomy and function are significantly impacted both during and after release of obstruction, and the degree of change is greatly influenced by whether the obstruction is unilateral or bilateral, acute or chronic, and partial or complete. Most mechanistic studies on UO-related renal dysfunction use experimental models of acute complete unilateral (UUO) or bilateral (BUO) ureteral obstruction, usually for 24 h. In these “classical” animal models, the extent of UO is clear and reproducible, and the obtained results are not confounded by extensive inflammation- or fibrosis-induced changes in the renal parenchyma; the BUO model especially is often used to explore the pathophysiological regulation of transport proteins (23–25). Complete obstruction (UUO/BUO) for a short duration strikingly alters renal blood flow (RBF), glomerular filtration, and tubular functions but produces minimal anatomical changes in blood vessels, glomeruli, and tubules (6). The responses to prolonged obstruction following complete UUO include interstitial inflammation (peak at day 2–3), tubular dilation and atrophy, as well as tubulointerstitial fibrosis (TIF), which is mostly observable from day 7 onward (26). However, the direct translation of results from these models has limitations and leaves a gap of knowledge compared with the chronic disease development of CKD as a consequence of obstruction in humans. Because most cases of clinical UO involve partial rather than complete obstruction, animal models of partial UUO have been established in both neonatal and adult mice and rats (27, 28). These models can be used to study progressive renal injury and TIF (29); however, the partial UUO model is technically very challenging, with frequent adhesions leading to complete obstruction. As detailed in this review, paramount knowledge of the pathophysiology has been obtained from these experimental models of urinary tract obstruction. However it is important to underline that all these animal models of obstructive nephropathy have limitations for direct comparison of the pathophysiological changes in humans with chronic obstruction of the urinary tract.

### 3.2. The UUO Model—Strengths and Limitations

UUO is the standard experimental model for TIF because it is not complicated by hypertension, proteinuria, hyperlipidemia, or toxicity-driven renal injury (30–32). UUO has emerged as a central model to provide information on myofibroblast activation and extracellular matrix (ECM) accumulation in the context of renal TIF (26, 33), and the model recapitulates fundamental pathophysiological processes that typify human CKD in an accelerated time span (4, 34), although the model misses important aspects of tubular repair.

The major advantages of the UUO model are that it is easy to perform and fast (generally, experiments last for 3–14 days) and it has a high degree of reproducibility, even in mice. Moreover, the model can be modulated with respect to timing, severity, and duration of the obstruction. However, even though UUO is one of the most popular models of CKD, it has limitations to take into consideration. One major disadvantage is the inability to precisely monitor changes in renal function. The contralateral nonobstructed kidney compensates for the loss of function in the obstructed kidney, so the levels of endogenous filtration markers, e.g., plasma creatinine and blood urea nitrogen (BUN), which are often used to assess renal function, hardly change. Moreover, in the UUO model there is no tubular recovery, so it is not an ideal model to study tubular injury and repair. Also, the precise cytopathological mechanisms driving UUO-induced renal injury are poorly understood (35). In addition, fibrosis develops very rapidly in the model, which limits the experimental window for testing potential ameliorating interventions unless they are introduced before the UUO injury or are expected to have profound effects (36, 37). Nevertheless, numerous putative antifibrotic compounds have shown therapeutic potential in this model (32), including dapagliflozin and empagliflozin (38, 39), which are both approved by the FDA for the treatment of CKD, as well as pirfenidone and nintedanib (40, 41), which are both approved by the FDA for the treatment of idiopathic pulmonary fibrosis. Another caveat of the UUO model, which is rarely discussed, is suture placement. This is an important variable because localization of the suture will affect the time course of renal injury and variation in placement can lead to problems of reproducibility.

Whether the UUO model can be used to study the structural and functional recovery of the kidneys after relief of the obstruction is controversial. Some studies have demonstrated that the obstructed kidney is able to regenerate after reversal of the obstruction (42–45), whereas others observed that renal damage persists after relief of obstruction (46–49). However, on the basis of the above-mentioned studies, it appears that there are three important aspects for achieving a successful reversible UUO model: *1*) optimal timing of the relief to allow for recovery; *2*) ability to achieve complete relief of the obstruction; and *3*) removing the contralateral kidney to avoid compensatory functional changes in the nonobstructed kidney (50). Overall, the reversible UUO model, although technically challenging, is considered to be an appropriate model to explore the process of tissue remodeling in the ipsilateral kidney. Moreover, the model can be of clinical relevance, as it reflects the pathophysiology of patients with UO who received decompression but are still at risk for ESRD due to persistent impairment of renal functions (51, 52).

### 3.3. Translation to Human CKD

For decades, the UUO model has been instrumental in preclinical research, and it is widely used to characterize the pathophysiology of CKD and test potential interventions. However, therapeutic strategies with proven efficacy in the UUO model cannot always be successfully translated into clinical practice because of several limitations: First, there are significant differences in physiology and pathophysiology between animals and humans, in particular because humans rarely present a picture with complete obstruction. Second, animal models do not reflect the variability of the patient population, since it is very common to choose a single inbred strain of animal of a single sex and with a specific age to minimize variance in animal experiments. Third, there is often a mismatch between the measured outcomes in preclinical research compared with clinical trials. To illustrate this, in UUO animals biochemical and histological parameters are mainly evaluated, whereas in humans more restricted end points such as GFR, proteinuria, or mortality are often employed. Fourth, and last, the UUO model does not reflect established disease, which is mostly seen in clinical practice. Therefore, it is paramount to validate findings in other CKD models, especially with regard to fibrosis progression. Moreover, there is a need for better animal models that closely resemble AKI-CKD transition (53). Despite these limitations, careful use of the UUO model can provide clinically relevant insight into the different pathophysiological mechanisms underlying CKD development and can unveil possible therapeutic targets.

## 4. FUNCTIONAL CHANGES IN OBSTRUCTIVE NEPHROPATHY

### 4.1. Hemodynamic Changes Associated with Obstructive Nephropathy

**Figure 3.**
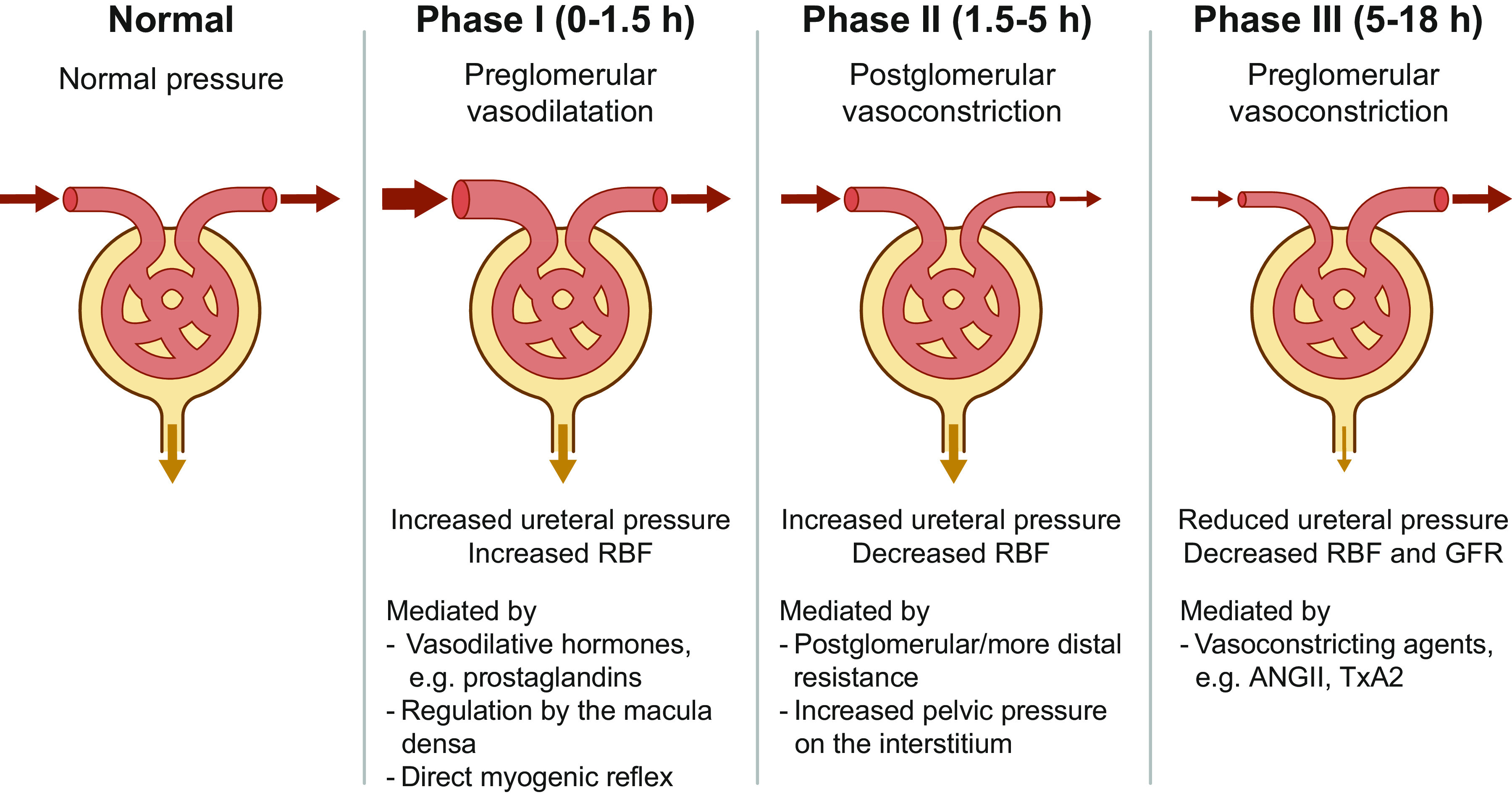
Characteristic phases of hemodynamic changes associated with obstructive nephropathy. ANG II, angiotensin II; GFR, glomerular filtration rate; RBF, renal blood flow; TxA_2_, thromboxane A2.

Obstructive nephropathy is characterized by severe vasoconstriction and profoundly changes all components of glomerular function. The extent to which glomerular function is affected depends on the duration and severity of the obstruction, whether it is unilateral or bilateral, and the extent to which the obstruction is persistent or has been relieved (54, 55). The hemodynamic changes can be divided into an early vasodilation phase (1–2 h of unilateral obstruction) and a late vasoconstrictive phase (>3 h of unilateral obstruction), as illustrated in FIGURE 3.

#### 4.1.1. The early vasodilation phase of obstruction.

Moody et al. (56) demonstrated a direct relationship between the hydrodynamic and hemodynamic events in the ipsilateral kidney after acute UUO for 18 h in awake dogs. Three distinct phases follow after the onset of UO: phase I (0–1.5 h), characterized by an increase in both ureteral pressure and RBF, possibly due to a predominant preglomerular vasodilation; phase II (1.5–5 h), characterized by an increase in ureteral pressure and a decrease in RBF, explained by postglomerular vasoconstriction; and phase III (5–18 h), characterized by a reduction in both ureteral pressure and RBF, explained by a predominant preglomerular vasoconstriction. The initial transient increase in RBF in phase I has been demonstrated in many studies in dogs (56–58), rabbits (59), rats (60), and pigs (61). This afferent vasodilation is mediated by several mechanisms, including increases in vasodilator hormones, such as prostaglandins (see below), reduced NaCl concentration at the macula densa, and a direct myogenic reflex (62). The hyperemic response is not attenuated by renal nerve stimulation or infusion of catecholamines (63, 64) and may be linked to changes in interstitial pressure (65, 66).

The increase in RBF after the onset of UO is not a consistent finding in all models. RBF has been reported to remain constant or decline after the onset of UUO in awake dogs (67), rats (68), lambs (69), and pigs (70, 71), suggesting a complex balance between vasodilatory and vasoconstrictor mechanisms at this early time point after obstruction. Upon the onset of UO, a rapid increase in pelvic (ureter) pressure could increase the peritubular capillary pressure. A constant RBF in the face of an increasing peritubular capillary pressure is consistent with some degree of arteriolar vasodilation (72). Blood flow measurements revealed that the initial vasodilation is expected both in the cortex and in the medulla (73). The mechanisms underlying the changes in the pre- and postglomerular vascular resistance, after the onset of UO, are still not completely understood. However, infusion of indomethacin, a nonselective inhibitor of the cyclooxygenase enzymes (COX-1 and COX-2), dramatically reduced RBF during UUO in pigs (74), suggesting that vasodilatory prostaglandins (e.g., PGE_2_) might be involved in the increase or the maintenance of RBF during UUO.

#### 4.1.2. The late vasoconstrictive phase of obstruction.

After the brief vasodilatory phase I, ipsilateral renal blood flow progressively decreases during UUO [phases II and III (56)]. In phase II, although pelvic (ureteral) pressure does not reach the maximum levels, the reduction in RBF is already ongoing, suggesting that primarily the postglomerular vasculature is vasoconstricted. Thus, the underlying mechanisms in phase II could be a selective increase in the postglomerular or more distal efferent arteriolar resistance or a direct result of the increasing pelvic pressure on the interstitium increasing the peritubular capillary pressure (56, 70, 71).

In phase III, RBF continues to decline and decreases by ∼30% of the preobstructive levels, with high plasma angiotensin II (ANG II) levels after 15 h of UUO (70), consistent with other studies demonstrating continued active vasoconstriction (56, 57). The mechanism underlying phase III is supposed to be the continuous vasoconstriction, localized predominantly at the preglomerular vasculature (67, 75). This finding is supported by micropuncture studies of individual surface glomeruli in the rat kidneys after blockade of tubules (76, 77). Consistent with this, UUO in rats for 24 h markedly increased the afferent arteriolar resistance and decreased afferent arteriolar blood flow (77). These studies highlighted the critical role of afferent arteriolar vasoconstriction in attenuating single-nephron GFR (SNGFR) during the established phase of obstruction and that of intrarenal mechanisms responsible for the late preglomerular vasoconstrictive response to UUO. Simultaneous with the progressive reduction in RBF, GFR, and renal tissue perfusion, oxygen delivery to the renal cortex and particularly to the renal medulla is reduced, leading to a gradual loss of the capillary network in the kidney, increased apoptosis, and stimulation of cell infiltration in the renal parenchyma (6). Numerous intrarenal vasoactive compounds, such as ANG II and thromboxane A_2_ (TxA2), are potentially implicated in the active vasoconstriction and subsequent stimulation of cellular and molecular processes, which involve inflammation, deposition of matrix proteins, and development of progressive interstitial fibrosis. Multiple studies revealed that several vasoactive mediators play significant roles in the reduction in RBF and GFR because prostaglandin E_2_ blockade, COX-2 inhibition (74), angiotensin II-converting enzyme (ACE) inhibitors (78–80), angiotensin II receptor blockade (81), inhibition of TxA2 synthesis (82), kallikrein inhibitor (83), and blockade of endothelin ET_A_ receptor (84) changed the RBF and GFR to some extent upon release of the obstruction. The role of these mediators has been highlighted in detail in a previous review (4).

#### 4.1.3. The renin-angiotensin-aldosterone system in the regulation of renal hemodynamics after ureteral obstruction.

The reduction in RBF in UUO is primarily the consequence of active vasoconstriction (57) and can be attenuated by specific pharmacological agonists or antagonists (85). Several such mediators are vasoconstricting agents, such as ANG II and TxA2; the changes in RBF in UUO in response to the blockade of ANG II or vasoactive eicosanoid prostaglandin system have been examined in several studies (74, 78, 81). Hammad et al. (86) demonstrated that ANG II infusion decreased RBF, GFR, and urine volume in rats. Vander and Miller (87) reported an increase in renin concentration in the renal vein when ureteral pressure was elevated. These findings evoked interest in the renin-angiotensin system (RAS) to understand the underlying pathophysiology of obstructive nephropathy as well as to introduce therapeutic interventions. Consistently, several studies demonstrated that administration of angiotensin-converting enzyme (ACE) inhibitor, captopril or enalapril, attenuated renal vasoconstriction during UO and improved postobstructive renal dysfunction in rat and guinea pig (79, 80, 88). In contrast, no significant improvement in postobstructive renal dysfunction was observed in UUO rats infused with an ANG II antagonist, saralasin, which might be partly attributable to vasoconstrictor properties of saralasin (78). Other investigators, however, demonstrated that ACE inhibitors did not decrease renal vasoconstriction in pigs (89). Infusion of an ACE inhibitor, captopril, in UUO pigs resulted in an increase in contralateral RBF, suggesting that a compensatory increase in RBF may in part be inhibited by ANG II-mediated vasoconstriction in the contralateral kidney during obstruction (89). These results are contrary to the notion that vasoconstriction in UUO is exclusively dependent on ANG II and suggest the role of other mediators in the vasoconstriction observed in obstructive nephropathy.

#### 4.1.4. The prostaglandin system in the regulation of renal hemodynamics after ureteral obstruction.

Administration of indomethacin during UUO decreased RBF in both the ipsilateral and contralateral kidneys. Simultaneously, a decrease in the urinary excretion of PGE_2_ from the contralateral kidney was observed after UUO, demonstrating inhibition of PGE_2_ production in the kidney after indomethacin treatment (74, 90). The reduction in RBF is immediate after indomethacin administration, and the initial increase in RBF following the onset of UUO subsided (91). Thus, the vasodilatory prostaglandins (e.g., PGE_2_) likely play an important role in the maintenance of RBF during UUO (92, 93). Administration of nonselective COX inhibitors blocks both vasodilatory (PGE_2_) and vasoconstrictor arachidonic acid (TxA2) products. The maintenance of RBF during UUO could not be achieved upon suppression of vasodilatory prostaglandins. In contrast, inhibition of the thromboxane system with a COX inhibitor resulted in the preservation of renal function (79). Administration of aprotinin, a kallikrein inhibitor, also improved postobstructive function by decreasing kinin-stimulated production of TxA2 in rats subjected to 24-h UUO (83). These findings indicate multiple sites of action in the vasculature of obstructed kidneys by vasoactive prostanoids.

### 4.2. Changes in Urinary Concentration in Response to Obstructive Nephropathy

#### 4.2.1. Urinary concentration defect in ureteral obstruction.

**Figure 4.**
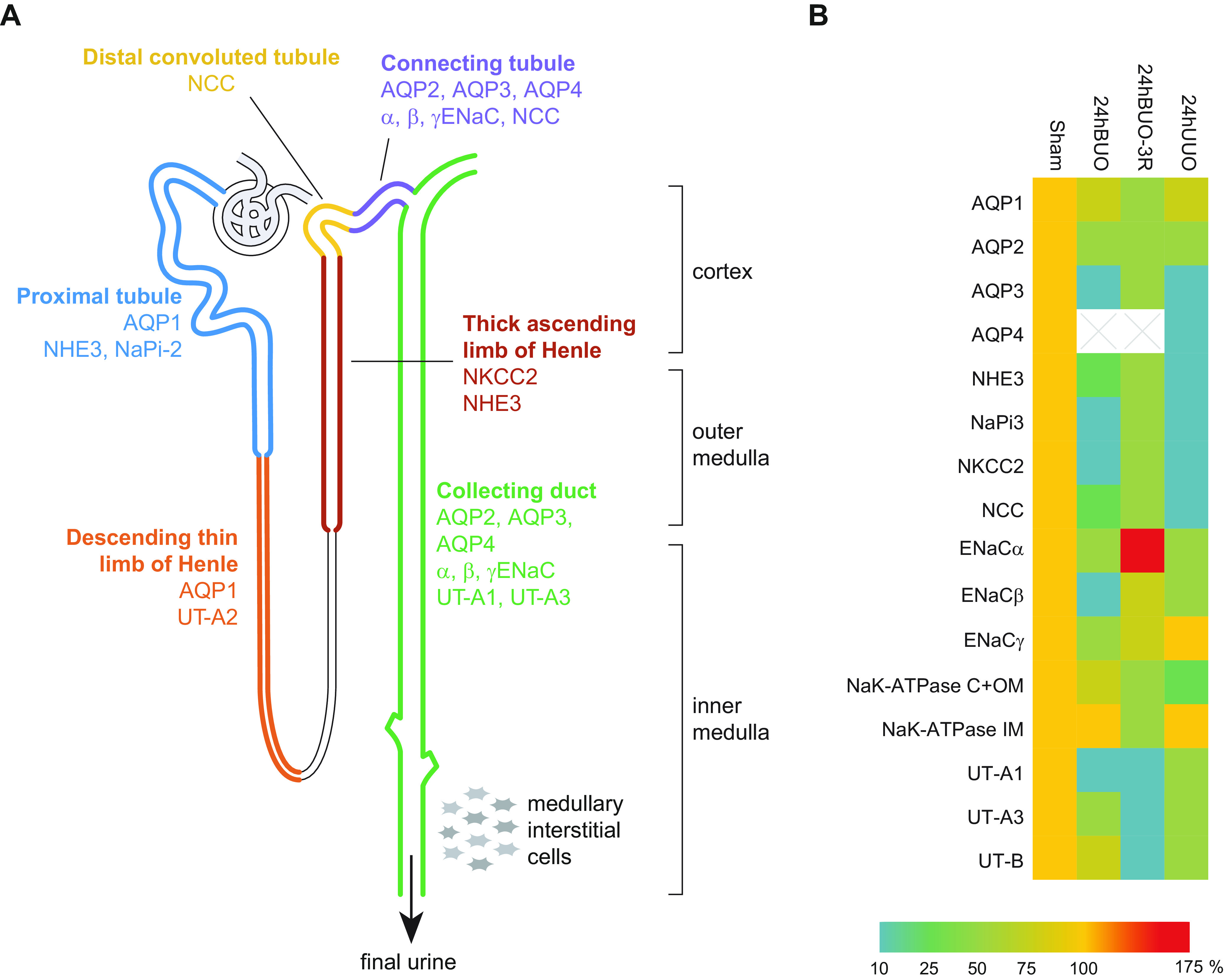
*A*: expression of renal water channel proteins (aquaporins), sodium transporters, and urea transporters (UTs) that contribute to tubular reabsorption of water, sodium, and urea in each segment of the nephron. *B*: changes in the expression of the aquaporins and sodium and urea transporters in different models of ureteral obstruction compared with the healthy situation (Sham, 100%), based on Refs. 24, 25, 96–99. 24hBUO, 24 h of bilateral ureteral obstruction; 24hBOU-3R, 24 h of bilateral ureteral obstruction followed by the release of obstruction for 3 h; 24hUUO, 24 h of unilateral ureteral obstruction; AQP, aquaporin; ENaC, epithelial sodium channel; NaPi-2, type II Na-Pi cotransporters; NCC, Na-Cl cotransporter; NHE3, type 3 Na/H exchanger.

Urinary tract obstruction is associated with changes in renal water handling. Studies using isolated perfused tubules from animal models of BUO and UUO, including proximal straight tubule, medullary thick ascending limb (TAL), and cortical collecting tubule, revealed marked impairment of fluid and chloride reabsorption capacity (94, 95). Consistently, altered expression of water channel proteins and sodium transporters in the kidney has been demonstrated in obstructive nephropathy (FIGURE 4). The expression of aquaporin-1 (AQP1) in the proximal tubule and descending thin limb and of AQP2, -3, and -4 in the collecting duct was significantly decreased in experimental BUO for 24 h (23, 24). Moreover, downregulation of sodium transporters [type 3 Na/H exchanger (NHE3), type II Na-Pi cotransporters (NaPi-2), Na-K-2Cl cotransporter (NKCC2), Na-Cl cotransporter (NCC), epithelial sodium channel (ENaC)], Na-K-ATPase and urea transporters (UT-A1, UT-A3, and UT-B) in BUO kidneys contributes to the impaired urinary concentration capacity (25, 96, 97). Postobstructive polyuria and decreased urine concentration follow the release of 24-h BUO. Li et al. (24) demonstrated that AQP2 and AQP3 levels in the collecting duct remained decreased for ∼2 wk after the release of 24-h BUO and were normalized 30 days after the release. AQP1 levels were also significantly reduced and remained downregulated for 30 days after the release (24), indicating that prolonged downregulation of AQPs in the renal tubules in obstructive nephropathy contributes to changes in renal water handling, that is, postobstructive polyuria and decreased urine concentration.

In contrast to BUO, UUO does not significantly change the total urinary excretion of water and sodium because the nonobstructed contralateral kidney compensates for the dysfunction of the obstructed kidney to excrete water and solutes (98, 100). Importantly, these studies demonstrated long-term parallel reduction in AQP2 expression and development of polyuria, decreased urine osmolality, and increased free water clearance, indicating a functional association between AQP2 abundance and the capacity to excrete free water (24, 98, 100). The levels of AQP1, -2, -3, and -4 and phosphorylated AQP2 at serine 256 as well as major sodium transporters (NHE3, NaPi-2, NKCC2, NCC, and ENaC) and urea transporters (UT-A1, UT-A3, and UT-B) were significantly decreased in the obstructed kidney in 24-h UUO (96–100) (FIGURE 4). The downregulation of AQP2 in obstructed kidneys was associated with reduced expression of AQP2 mRNA, suggesting that AQP2 was regulated at the transcriptional level (100). No systematic comparison between the UUO model and human CKD has been performed related to the regulation of different transport proteins. However, there are reports suggesting a similar reduction in protein expression of AQP2 and -3 in biopsies from human kidneys with substantial interstitial fibrosis and nephron loss (101).

#### 4.2.2. Underlying mechanisms responsible for the regulation of channels and transporters in response to ureteral obstruction.

BUO is associated with the induction of COX-2 and cellular infiltration in the renal medulla (102–105), which may contribute to the downregulation of AQP2. Consistently, local accumulation of COX-2-produced prostanoids (PGE_2_, PGF_2α_, 6-keto-PGF_1α_, PGD_2_, and thromboxane B_2_) was observed in BUO kidneys (104). UUO for 7 days was also associated with increased COX-1/2 and microsomal prostaglandin E synthase 1 levels in the kidney as well as PGE_2_ secretion in urine collected from the obstructed ureter (106). The infiltration of leukocytes following UO was associated with an increase in thromboxane B_2_ excretion by the kidney and coincided with a reduction in GFR (107). In contrast, treatment with parecoxib, a COX-2 inhibitor, attenuated the increase in the concentrations of PGE_2_, PGF_2α_, 6-keto-PGF_1α_, and thromboxane B_2_ in the inner medulla of 24-h BUO kidneys (104). Importantly, COX-2 inhibition in rats with 24-h BUO significantly attenuated the downregulation of AQP2 in the inner medulla and of sodium transporters (NHE3 and NKCC2) in the proximal tubule and medullary TAL (103). These findings indicate that COX-2 and associated prostanoids regulate, at least in part, the expression of water channel proteins and sodium transporters, which play a role in impaired renal water and sodium handling in response to BUO (103, 108, 109).

Renin mRNA levels and ANG II content in the kidneys increase in obstructive nephropathy in rats, coinciding with high systolic blood pressure and plasma ANG II levels (110). Notably, treatment of BUO rats with candesartan, an ANG II type 1 (AT1) receptor blocker, significantly attenuated the downregulation of NaPi-2 in the proximal tubule, NKCC2 in the medullary TAL, and AQP2 in the collecting duct at 2 days after the release of 24-h BUO (111). Moreover, AT1 receptor blockade attenuated the increased expression of COX-2 in the inner medulla, suggesting that COX-2 induction after BUO and related changes in the expression of sodium transporters and AQP2 could be regulated, at least in part, by ANG II (111). These observations were different from the findings in normal rats because ANG II per se potentiates the effect of vasopressin on the plasma membrane targeting of AQP2 (112) and regulates the expression of AQPs and sodium transporters in the renal tubule (113–115).

Consistent with the COX-2 induction observed in the kidney inner medulla in BUO, COX-2 mRNA and protein levels are significantly increased in the inner medulla in response to UUO (116, 117). The role of reactive oxygen species (ROS) in the induction of COX-2 was demonstrated in rats with 3-day UUO: antioxidants (NADPH oxidase inhibitor diphenyleneiodonium and the complex I inhibitor rotenone) decreased the UUO-mediated induction of COX-2 in the kidney inner medulla (118). Treatment with superoxide dismutase 2 (SOD2)-mimic manganese(III)tetrakis[(4-benzoic acid) porphyrin chloride (MnTBAP)] also significantly attenuated AQP2 downregulation in 7-day UUO and suppressed COX-2 induction in the kidney (106). This finding suggested that mitochondrial oxidative stress partly mediates AQP2 downregulation in obstructive nephropathy through the COX-2 pathway. However, the underlying mechanisms and signaling pathways responsible for the downregulation of channels and transporters in the renal tubule in response to UO are complex. Other possible factors include high hydrostatic pressure on tubular epithelial cells, increased interstitial pressure, changes in blood circulation in the kidney, absence of urine flow, production of natriuretic substances, and altered expression of genes associated with cell transformation/transition. Indeed, the effects of changes in the microenvironment, such as osmolality, urine flow, and pH, on the expression of AQP2 were demonstrated (119–121).

## 5. METABOLIC CHANGES IN OBSTRUCTIVE NEPHROPATHY

UO is associated with significant changes in metabolism that coincide with tubular cell injury and macrophage infiltration in the interstitium (26). Metabolites are endogenous and exogenous molecules that play a role in regulatory systems in the cells, and metabolic profiling has been performed to understand the metabolic responses of kidney cells under physiological and pathophysiological conditions (122–127).

**Figure 5.**
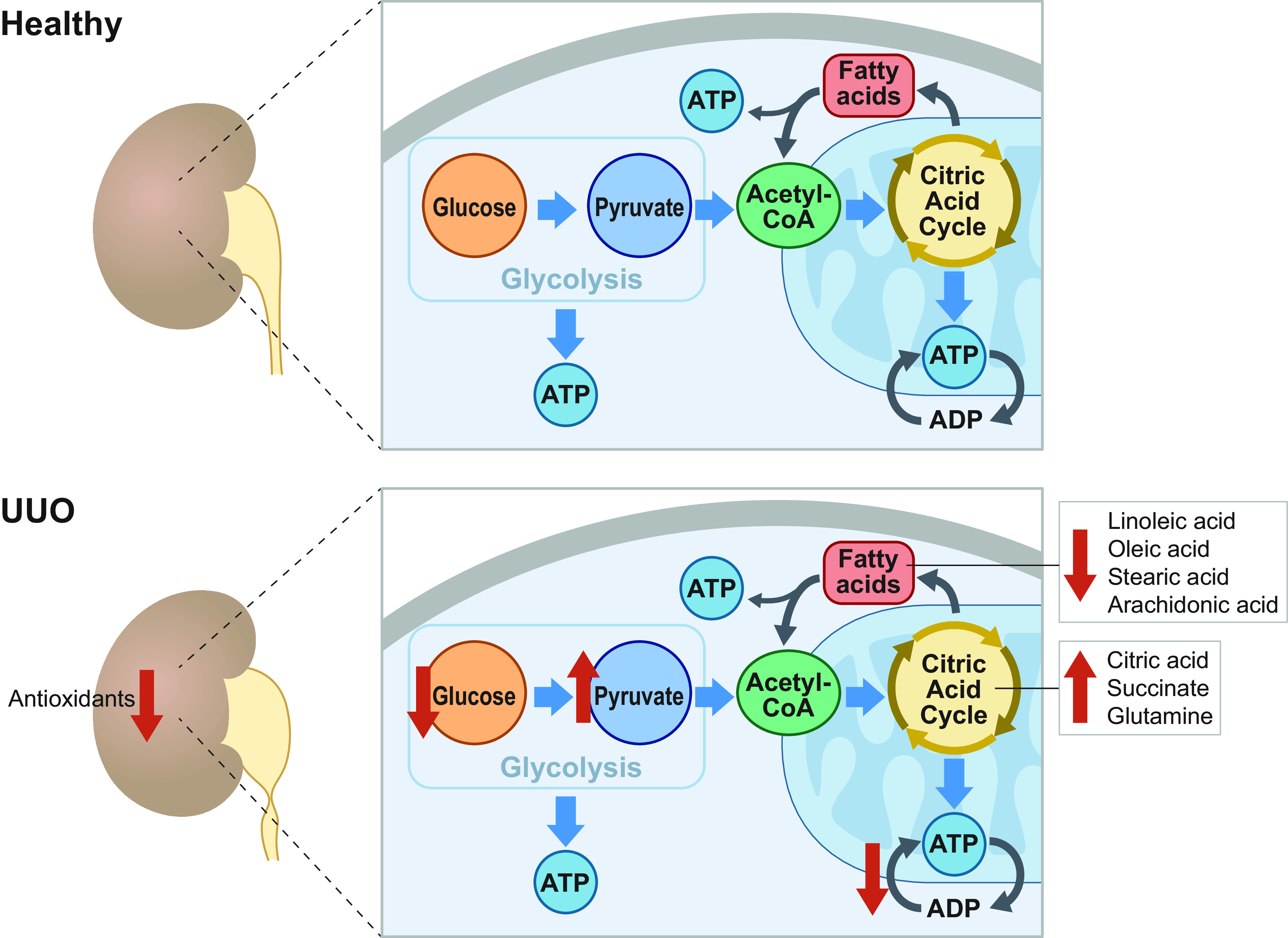
Overview of the renal metabolic changes in response to 1 and 3 wk of unilateral ureteral obstruction (UUO). Because of the myriad of tasks performed by the kidney to maintain total body homeostasis, it is one of the most energy-demanding organs in the body. In the healthy kidney, energy, in the form of ATP, is generated via glycolysis, in the citric acid cycle, and via β-oxidation of fatty acids. In obstructive nephropathy, ATP production is severely impaired because of *1*) perturbations in glycolysis, resulting in decreased levels of glucose and increased levels of pyruvate; *2*) interruption of the citric acid cycle, causing accumulation of citric acid, succinate, and glutamine; *3*) changes in ATP metabolism; and *4*) impairment of fatty acid metabolism, leading to a reduction in the levels of linoleic acid, oleic acid, stearic acid, and arachidonic acid. Moreover, there is an overall reduction in renal antioxidant systems. These metabolic changes are believed to promote fibrogenesis following UUO.

Mass spectrometry imaging of the rat kidneys after 1 and 3 wk of UUO revealed altered renal metabolism, such as glycolysis (decreased levels of glucose and increased levels of pyruvic acid), tricarboxylic acid cycle (increased levels of citric acid, succinate, and glutamine and decreased level of aspartate), ATP metabolism (reduced ADP and AMP levels), fatty acid metabolism (reduced levels of linoleic acid, oleic acid, stearic acid, and arachidonic acid), antioxidants (reduced levels of taurine and GSH), and electrolytes (reduced levels of Na^+^ and K^+^) (128) (FIGURE 5). Moreover, a marked increase in triglyceride content and a decrease in total phospholipid content were observed in kidneys of rats subjected to 24 h of UUO (129). These changes in renal metabolism in UUO could contribute to the development of renal fibrosis. Hyperpolarized ^13^C magnetic resonance imaging revealed an increase in the lactate-to-pyruvate ratio in the kidneys of mice after partial UUO (130). Notably, these changes were associated with hydronephrosis, fibrosis, and macrophage infiltration in the obstructed kidney (130). In animal models, a metabolic shift from mitochondrial oxidative phosphorylation to aerobic glycolysis was observed in renal fibrosis (128, 131, 132).

In several studies, kidney tissue and fibroblasts exhibited increased expression of mRNA and protein levels of glycolytic enzymes [hexokinase (HK2), pyruvate kinase M2 (PKM2), lactate dehydrogenase A (LDHA)] and lactic acid production in the process of fibrosis (132–134). Importantly, shikonin (an inhibitor of PKM2) and dichloroacetate [an inhibitor of pyruvate dehydrogenase kinase-1 (PDK1)] significantly inhibited the expression of fibronectin and type I collagen, tubular apoptosis, and macrophage infiltration in the obstructed kidney in a mouse model of 7-day UUO (135). In line with this, it was found that shikonin and 2-deoxyglucose, another aerobic glycolysis inhibitor, attenuated myofibroblast activation in UUO-induced fibrosis. Moreover, it was shown that shikonin inhibits aerobic glycolysis by reducing phosphorylation of PKM2, the rate-limiting glycolytic enzyme associated with cell reliance on aerobic glycolysis (136). However, another study of the 7-day UUO model revealed that only very few regulators of glucose utilization were differentially expressed in the obstructed kidneys (137). Clearly, more studies are needed to examine the changes in the protein levels and activity of each enzyme involved in the whole glycolytic pathway in the obstructed kidney. Mitochondrial fatty acid β-oxidation of long-chain fatty acids, in which carnitine palmitoyltransferase 1 (CPT1) is the critical regulatory enzyme (138), provides energy to renal tubular epithelial cells (139), and decreased fatty acid oxidation is associated with renal fibrosis (140, 141). Indeed, in carnitine palmitoyl-transferase 1A (Cpt1a)-knockin mice overexpression of Cpt1a decreased renal fibrosis, blunted proinflammatory responses, inhibited epithelial cell damage and macrophage infiltration, and prevented mitochondrial dysfunction (137). Thus, the metabolic alterations in renal fibrosis, including changes in glucose and lipid metabolism, are important pathophysiological features that potentially could become important therapeutic targets (142–145).

## 6. TRANSCRIPTIONAL CHANGES IN OBSTRUCTIVE NEPHROPATHY

Obstructive nephropathy is associated with tubular apoptosis, interstitial inflammation, and interstitial fibrosis, and there are complex changes in gene expression and protein abundance. The observed changes could serve as potential biomarkers of disease progression and therapeutic targets. These include signaling molecules involved in macrophage recruitment and proliferation, tubular cell activation/differentiation, cell adhesion, apoptosis, immune/inflammatory responses, cell cycle regulation, proteolysis, metabolic processes, transport functions, and epithelial-mesenchymal transition (EMT) (146–148). Microarray analysis of obstructed kidneys of rats with neonatal UUO revealed that mRNA expression of multiple immune modulators, including Krox24, interferon-γ regulating factor-1 (IRF-1), monocyte chemoattractant protein-1 (MCP-1), interleukin-1β (IL-1β), CCAAT/enhancer binding protein (C/EBP), p21, c-fos, c-jun, and pJunB, was significantly upregulated compared with sham-operated kidneys on day 12 after obstruction (149). Moreover, microarray analysis of mouse kidneys subjected to UUO revealed that >1,800 transcripts were changed during days 1 through 9 after obstruction (147). These changes include many transcripts, such as Fos protooncogene, AP-1 transcription factor subunit (FOS), CD44, clusterin (CLU), secreted phosphoprotein 1 (SPP1), and epidermal growth factor (EGF). Pathway analysis showed that upregulated transcripts were enriched in cell cycle/chromosome segregation, organization, immune/inflammatory responses, cell adhesion, cell activation/differentiation, and development terms. In contrast, downregulated transcripts were mainly involved in metabolic processes, transport functions, and oxidation-reduction (147). Network analysis using the Ingenuity Pathway Analysis (IPA) software showed that CCAAT/enhancer binding protein-β (CEBPB) and hepatocyte nuclear factor 4-α (HNF4A) played a central role in the transcriptional regulation of genes implicated in UO or renal fibrosis (147). Higgins et al. (150) identified differentially expressed genes in obstructed kidneys of C57BL/6 mice on days 4 and 10 after UUO, using Affymetrix microarrays. Several profibrogenic genes, whose mRNA levels were upregulated in obstructed kidneys after UUO, were fibroblast-inducible secreted protein, murine homolog of connective tissue growth factor, collagen XVIII alpha1, src-suppressed C-kinase substrate, and secreted protein acidic and rich in cysteine (SPARC) (150). Consistent with this, Gerarduzzi et al. (151) demonstrated that silencing secreted modular calcium-binding protein 2 (SMOC2), which belongs to the SPARC family, attenuated renal fibrosis by inhibiting transforming growth factor-β (TGFβ)-mediated fibroblast-to-myofibroblast transition.

RNA-seq technology has recently provided novel insights into gene expression and regulatory networks. In a mice model of UUO (2 days or 8 days after ligation), RNA-seq analysis revealed change in the expression of thousands of genes (148). Many novel protein-coding genes were identified, including many upregulated genes, such as *Fblim1*, *Epha2*, *Wisp1*, *Parvg*, *Madcam1*, *Mybpc2*, *Cdh3*, *Gpr56*, *Troap*, *Pstpip1*, *Pcdh8*, *Nlgn2*, and *Mfap4*, potentially associated with renal fibrosis. In addition, many critical transcription factors that could potentially be implicated in renal fibrosis, such as Sox9, Runx1, Uhrf1, and Ezh2, were upregulated. Several other critical transcription factors, including Fosl1, Fosl2, Fos, Jun, JunD, Egr2, Creb5, FoxJ1, Ikzf4, Atf5, Sox4, and Sox11, were also upregulated. p53 (*Tp53*) is also an important transcriptional coregulator of several TGFβ fibrotic-response genes (31). The contribution of all these transcription factors is still not completely understood with regard to their selective role in UUO-induced fibrosis. Moreover, long noncoding RNAs (lncRNAs) are differentially expressed in the mouse model of UUO, which is likely to affect the expression of fibrosis-related proteins and the cellular phenotype (148). In particular, lncRNAs TCONS_00088786 and TCONS_01496394 were regulated by TGFβ stimulation, and they can affect the expression of fibrosis-related genes through a feedback loop (152). Pavkovic et al. (153) used a comprehensive and combined multiomics data set (mRNAs, proteins, and microRNAs) in UUO mice to understand the molecular pathogenesis of kidney fibrosis. α-Smooth muscle actin (Acta2), collagen (Col1a1), and fibronectin (Fn1), which are fibrotic markers, were constantly increased over time (days 3, 7, and 14 after ligation) in the irreversible UUO model. Kidney injury markers, namely clusterin (Clu), kidney injury molecule 1 (Kim-1), and lipocalin-2 (Ngal), were increased early on without further significant increases over time in the obstructed kidneys. miR-192 was decreased, whereas miR-21 was increased, in obstructed kidneys in the UUO model. Further studies are warranted to elucidate the role of up- or downregulated transcripts in obstructed kidneys, particularly for new therapeutic approaches to renal fibrosis.

## 7. CELLULAR CHANGES IN OBSTRUCTIVE NEPHROPATHY

Surgical blockade of the urinary flow results in increased hydrostatic pressure, which initially affects the collecting ducts and then rapidly expands to the distal and proximal tubules (32). Long-term UUO causes outer medullary ablation and atrophy of the tubules. Within 14 days of UUO, the proximal tubule mass can decrease up to 65% and epithelial apoptosis/necrosis, infiltration of inflammatory cells, interstitial expansion with increased cell proliferation, as well as subsequent fibrosis are prominent in the cortical compartment of the obstructed kidney (30, 154). In addition, >80% of glomeruli were distinctly transformed after 14 days of UUO and displayed atubular glomeruli, indicating that the glomerulotubular junction is particularly vulnerable to UUO-induced damage (154).

### 7.1. Role of Glomeruli and Proximal Tubules in Obstructive Nephropathy

During the middle of the twentieth century, the glomerular and tubular epithelium received increased attention as primary targets of progressive renal damage (53, 154, 155). Progressive development of atubular glomeruli apparently plays an important role in obstructive nephropathy. An atubular glomerulus is one that is not connected to its proximal tubule, and this glomerulotubular disconnection is observed in many tubulointerstitial disorders. The presence of atubular glomeruli in diseased kidneys was originally described by Jean Oliver back in the 1930s (156). On the basis of sophisticated microdissection studies, Oliver demonstrated the presence of glomerulotubular disconnection in kidneys from patients with chronic Bright’s disease, which is now described as chronic glomerulonephritis. Subsequently, Marcussen published a series of articles (157–159) in the 1990s highlighting the importance of atubular glomeruli in a variety of patients with CKD due to different forms of renal diseases. He used serial sections rather than microdissection to demonstrate the discontinuity between glomeruli and tubules and reported that the percentage of atubular glomeruli in advanced cases of CKD may reach >35% (158).

The first description of atubular glomeruli in relation to obstructive nephropathy was in dogs subjected to UUO in 1965 in which the glomeruli were perfused but were not able to filter, indicating a glomerulotubular disconnection (160). Moreover, Konda et al. (161) reported an increase in renin-containing cells in the juxtaglomerular apparatus of atubular glomeruli in children with obstructive nephropathy. The presence of atubular glomeruli in fibrotic areas indicated that these areas may contribute to increased production of renin as well as of ANG II, which may promote progressive renal scarring. Forbes et al. (154) used histomorphometry to investigate the formation of atubular glomeruli in adult mice subjected to complete UUO. They showed that destruction of the glomerulotubular junction and formation of atubular glomeruli developed in 80% of nephrons after only 14 days of UUO. The glomerulotubular junction became atrophic because of autophagy and apoptosis along with concurrent remodeling of Bowman’s capsule to form atubular glomeruli. In this deterioration, epithelial cells in the urinary pole underwent transition to a mesenchymal phenotype and expanded to cover the capsule. At the same time, perfusion of the atubular glomeruli was maintained, although renin-positive cells were significantly increased along the afferent arterioles (154).

In line with this, Chevalier (53) highlights the proximal tubules as a primary sensor and effector in the progression of CKD as well as AKI. Because of its high rates of oxygen consumption and relative lack of endogenous antioxidant defenses, the proximal tubule is particularly vulnerable to injury. Interestingly, clinical studies of kidney injury reveal that targeting of the proximal tubule is adequate to induce AKI that can progress to CKD when the injury is repeated, indicating the proximal tubules as the central initiator of AKI-CKD progression. Another factor promoting renal injury in both AKI and CKD is the loss of tubular secretion (162), which can result in the accumulation of endogenous and exogenous waste products, including uremic toxins (163, 164). In CKD patients, elevated serum levels of uremic toxins are associated with an increased risk of adverse events, including cardiovascular mortality (165–167). It is also reported that uremic toxins accumulate and worsen the prognosis in AKI patients (168, 169). Interestingly, it was recently demonstrated that the plasma levels of uremic toxins were increased in 14-day UUO rats and positively correlated with TIF (170). The precise molecular mechanisms underlying uremic toxicity remain to be elucidated; however, it is clear that uremic toxins can act directly on various cell types, including proximal tubule cells (171, 172).

Taken together, these events indicate that both degenerative and regenerative processes are activated in response to the formation of atubular glomeruli following UUO and that the glomerular and tubular epithelium play an important role in progressive renal injury.

### 7.2. Tubulointerstitial Fibrosis

As mentioned above, a series of events is triggered after UUO, resulting in altered hemodynamics, changes in glomerular filtration, and architectural malformations (e.g., hydronephrosis); therefore, it is difficult to identify the earliest molecular or cellular response that provides the initial trigger for the ensuing fibrosis. Presumably, the increase in tubular pressure and mechanical stress results in tubular epithelial injury and interstitial macrophage infiltration, which in turn causes activation of resident fibroblasts and cellular differentiation into myofibroblasts, which are the main producers of ECM.

Fibrosis is characterized by the excessive deposition of ECM proteins, such as collagens and fibronectin, mainly by activated myofibroblasts. Irrespective of etiology, the fibrosis ultimately results in the loss of organ architecture and function, necessitating renal replacement therapy or transplantation. Most fibrosing renal diseases originate in the tubules, resulting in TIF.

### 7.3. Tubulointerstitial Fibrosis and the Origin of Myofibroblasts

**Figure 6.**
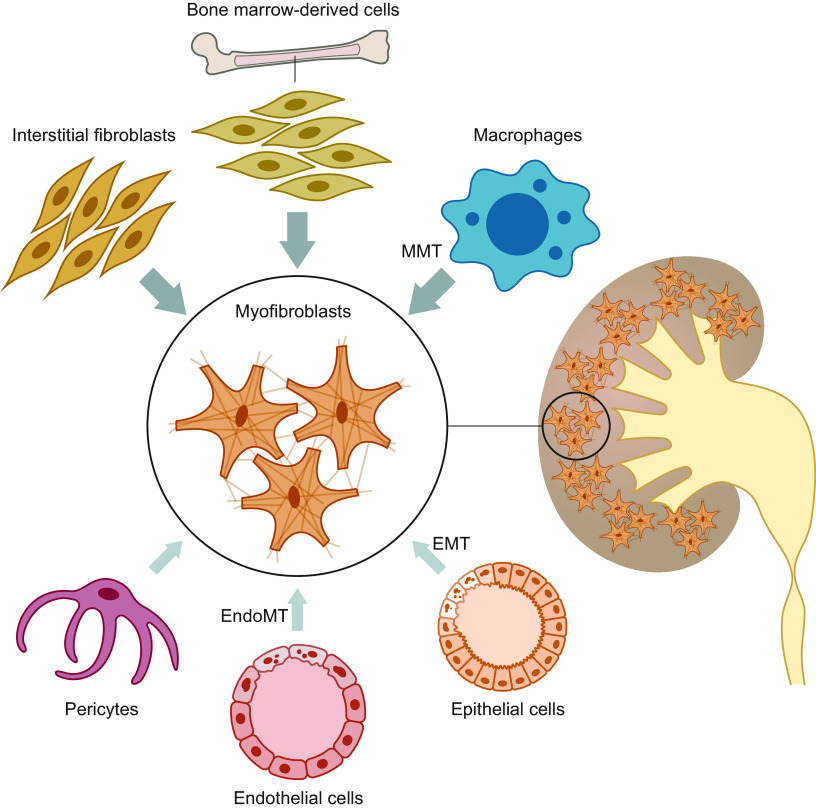
To date, resident fibroblasts and hematopoietic cells migrating into the renal tissue are considered the most important progenitors of collagen-producing myofibroblasts, whereas epithelial and endothelial cells contribute to a lesser extent to the myofibroblast pool. EMT, epithelial-to-mesenchymal transition; EndoMT, endothelial-to-mesenchymal transition; MMT, macrophage-to-myofibroblast transition.

This section describes the different origins of myofibroblasts, including interstitial resident fibroblasts, bone marrow-derived cells, tubular epithelial cells, endothelial cells, pericytes, and macrophages (FIGURE 6).

#### 7.3.1. Role of interstitial fibroblasts in TIF.

Until the 1990s, it was generally accepted that myofibroblasts arose from local resident kidney fibroblasts (173, 174). Interstitial resident fibroblasts can be detected by electron and fluorescence microscopy based on strong expression of ecto-5′-nucleotidase (CD73) in their plasma membrane (175–177). Picard and colleagues (178) investigated the alterations in resident fibroblasts upon conversion into myofibroblasts during the first 4 days of UUO. Using double immunofluorescence, they observed the expression of the myofibroblast marker α-smooth muscle actin (αSMA) in ecto-5′-nucleotidase-positive cells already on the first day after UUO, and this coexpression became progressively more frequent up to day 4. Thus, it appears that during the first days of UUO the resident fibroblasts can acquire the phenotype of myofibroblasts, indicating that this phenotypic alteration of resident fibroblasts can occur at a very early stage after UUO. Certain subpopulations of resident fibroblasts can produce erythropoietin (EPO) (179). Asada and coworkers (180) demonstrated that EPO-producing resident fibroblasts from kidneys in 14-day UUO mice can transdifferentiate into αSMA-positive myofibroblasts, in the same way as other resident fibroblasts in renal tissue, at the cost of EPO production. Reversal of the UUO-induced injury restored the production of EPO and the physiological phenotype of EPO-producing cells, indicating that myofibroblasts maintain reversibility in response to improvement of the microenvironment (181). Moreover, LeBleu and colleagues (182) performed a comprehensive study in 2013 using >20 different genetically engineered mouse lines to address the question of myofibroblast linage in the UUO fibrosis model at days 2, 5, and 10 after UUO. They demonstrated that accumulation of myofibroblasts occurred predominantly from two primary sources: ∼50% were derived by local proliferation of resident interstitial fibroblasts, and ∼35% were derived from bone marrow cells without any evidence of proliferation in the kidney tissue (FIGURE 6).

#### 7.3.2. Role of bone marrow-derived cells and macrophages in TIF.

Bone marrow-derived macrophages and monocytes play an important role in the pathophysiological processes following UUO. When describing these processes, the macrophage population is generally divided into two distinct groups with different functions, namely M1 and M2 macrophages. The classically activated M1 macrophages are regarded as proinflammatory, and alternatively activated M2 macrophages are considered profibrotic. Although this is a gross oversimplification, as delineated below, we use the same classification here. After obstruction, circulating classical monocytes (CD14^++^CD16^−^ in humans and Ly6C^hi^CCR2^hi^CX3CR1^lo^ in mice) (183) are recruited to the injured kidney, where they proliferate and acquire a proinflammatory M1 phenotype. Various chemokines, including C-C motif ligand (CCL)-2 (184), CCL5 (184), spleen tyrosine kinase (185), and osteopontin (186), are involved in the recruitment phase. Rovin and colleagues (187) demonstrated that macrophage infiltration peaks between 4 and 12 h after obstruction. M1 macrophages are predominant in the kidney up to day 3 after UUO, whereafter M2 macrophages become the dominant type (188). M1 polarization is induced by cytokines and danger signals, such as interferon (IFN)-γ, tumor necrosis factor (TNF)α, and granulocyte macrophage-colony stimulating factor (GM-CSF), present in the microenvironment of injured renal tissue (189). These M1 macrophages are procoagulant and produce cytokines (e.g., IL-1β, TNFα, and IL-6), oxygen radicals, and proteolytic enzymes [e.g., matrix metalloproteinase (MMP)-12] that not only enhance the inflammatory response but can also cause renal injury (190). The subsequent transition to an M2 phenotype is partly driven by tissue-type plasminogen activators (191). M2 macrophages (F4/80^+^CD206^+^) contribute to fibrosis by secreting profibrotic factors such as TGFβ (192). In addition, some older studies in which macrophage subpopulations were not differentiated showed that macrophages can promote fibrosis by expressing other factors, such as platelet-derived growth factor (PDGF)-C (193), galectin-3 (194), and MMP-9 (186).

As stated above, in vivo the macrophage population that is involved in UUO-induced renal injury is extremely heterogeneous. Using single-cell RNA sequencing (scRNAseq), Conway and colleagues (43) could identify 12 clusters of myeloid cells in a release of 7-day UUO (R-UUO) model, which were further divided into monocytes, macrophages, or dendritic cells (DCs). Monocytes could be classified as patrolling monocytes, proinflammatory Ly6C^+^ monocytes, and profibrotic Arg1^+^ monocytes. Interestingly, the latter were exclusively present on day 2 after UUO. Macrophages can be further classified as quiescent resident macrophages, Mrc1^+^ cells, which are closely aligned to embryonic macrophages; Ccr2^+^ macrophages, which are most likely derived from infiltrating Ly6C^+^ monocytes; IFN-induced macrophages; and Mmp12^+^ macrophages. Interestingly, the Mmp12^+^ macrophages represent a reparative phenotype, and these macrophages were solely present in kidneys that had undergone R-UUO. DCs could be categorized as type 1 and type 2 conventional DCs and lymph node DCs. Thus, it is clear that the population of immune cells involved in UUO comprises an extremely (functionally) diverse group of cells.

Moreover, bone marrow-derived circulating myofibroblast progenitors, either fibrocytes or M2 macrophages, can also contribute to renal fibrogenesis, but it is still unclear as to how much each of these cells contributes to the fibrosis. Fibrocyte-derived cells within the pool of collagen-producing cells have been estimated to range from nearly 0% to 50%, depending on the method used to identify these cells (182, 195–200). Additionally, it is unclear how many of the profibrotic M2 macrophages actually differentiate into collagen-producing myofibroblasts via a process termed “macrophage-to-myofibroblast transition” (MMT). Meng and colleagues (201) reported that in UUO mice as much as 65% of total αSMA^+^ myofibroblasts arose from MMT. Conversely, more recent studies have shown that only ∼10% of cells are F4/80^+^/αSMA^+^ (202–204), suggesting a modest contribution to the process of fibrosis. Importantly, in human fibrotic kidneys, only 0.01% of immune cells expressed both PDGFRβ and COL1α1 (205).

#### 7.3.3. Role of epithelial cells in TIF.

For decades, it was widely believed that EMT plays a key role in renal fibrogenesis. EMT is a process via which polarized epithelial cells acquire a mesenchymal phenotype. This phenotypical switch is characterized by the downregulation of epithelial markers, such as E-cadherin and zonula occludens-1, and upregulation of mesenchymal markers, such as αSMA and vimentin (FIGURE 6). These changes result in the cells exhibiting an enhanced capacity to migrate, resist apoptosis, and produce ECM components (206, 207). In 1995, Strutz et al. (208) postulated that during fibrogenesis the parenchymal epithelium might, at least in part, be converted into fibroblasts at the site of injury. This observation greatly stimulated research into the role of EMT in renal fibrosis; however, because of the absence of proper cellular markers, EMT remained a controversial topic (209). To overcome this hurdle, several research teams performed genetic fate-mapping studies to identify the progenitor cells of myofibroblasts in rodents subjected to UUO, and they demonstrated that only a minor fraction of collagen-producing myofibroblasts arose through EMT (182, 199, 210, 211). These findings were recently corroborated by Kuppe and coworkers. Using single-cell RNA sequencing (scRNAseq), they demonstrated that in human fibrotic kidneys only 0.28% of epithelial cells express both PDGFRβ and COL1α1, indicating that dedifferentiated proximal tubule cells hardly contribute to ECM production (205).

Although the majority of epithelial cells do not become ECM-producing fibroblasts, they do dedifferentiate upon injury and contribute to the fibrotic process. Recently, Wu and colleagues reported that after 14 days of UUO two distinct epithelial cell populations could be distinguished by scRNAseq: one was characterized by a strong proliferative gene signature next to increased expression of injury markers, such as Kim-1 (212), and the other population, consisting of dedifferentiated proximal tubule cells, was characterized by a strong cell movement genotype. Notably, these cells seem to be a source of proinflammatory cytokines, including CCL2 and IL-34 (212). In line with this observation, injured proximal tubule cells were reported to represent one of the main cellular sources of TGFβ signaling ligands to pericytes, fibroblasts, and myofibroblasts (205). These nonproliferative dedifferentiated epithelial cells also undergo metabolic reprogramming, as illustrated by the downregulation of genes related to solute transport, fatty acid metabolism, and β-oxidation (213). In particular, the loss of fatty acid oxidation appears to play a central role in renal fibrogenesis (214).

#### 7.3.4. Role of endothelial cells in TIF.

Endothelial-to-mesenchymal transition (EndoMT) is a process similar to EMT, wherein endothelial cells can undergo mesenchymal transition to form myofibroblasts (215–217) (FIGURE 6). In 2008, Zeisberg et al. (218) reported the contribution of EndoMT to renal fibrosis in mice subjected to 7-day UUO. They found that a considerable percentage (around 25–35%) of (myo)fibroblasts coexpress the endothelial marker CD31 and markers of fibroblast and myofibroblasts, such as FSP-1 and αSMA. Using an endothelial lineage-traceable transgenic mouse line (*Tier2-Cre; R26R-stop-EYFP*), they showed that endothelial cells labeled with yellow fluorescent protein (YFP) coexpressed FSP1 and αSMA, suggesting an endothelial origin of these fibroblasts. LeBleu et al. (182) demonstrated that ∼10% of myofibroblasts coexpressed the markers of endothelial cells and activated fibroblasts in lineage-tagged transgenic mice after 10-day UUO. Thus, these studies indicate that EndoMT may account for a substantial portion of myofibroblasts in UUO mice. However, in human fibrotic kidneys, only 0.16% of endothelial cells were shown to express both PDGFRβ and COL1α1 (205).

Recently, it was suggested that the soluble proteoglycan endothelial cell-specific molecule 1 (ESM1) may serve as an EndoMT marker of progression of renal fibrosis at day 7 and day 14 after UUO (219). In addition, Zhao and colleagues (220) showed that MMP-9, a major proteolytic enzyme, contributed to UUO-induced fibrosis via EndoMT. Deficiency of MMP-9 contributed to a 30% reduction in total αSMA-positive myofibroblasts arising from EndoMT of peritubular endothelial cells. However, these results need confirmation using lineage-tracing techniques. Although these studies provide some evidence of EndoMT in vivo, the essential role of EndoMT in renal fibrogenesis remains highly controversial.

#### 7.3.5. Role of pericytes in TIF.

Pericytes, which are ECM-producing cells localized in the subendothelial region, have been suggested to be another source of myofibroblasts (FIGURE 6). Lin et al. (217) and Humphreys et al. (210) identified pericytes and perivascular fibroblasts as myofibroblast precursors in the UUO model of kidney fibrosis. Kramann et al. (221) identified a group of Gli1-positive perivascular mesenchymal stem cell-like pericytes as myofibroblast progenitors using lineage tracing and cell ablation. Furthermore, using transgenic Gli-CreER^t2^;tdTomato 10-day UUO mice, they showed that Gli1-positive cells proliferate within the interstitial area and express αSMA, indicating perivascular Gli1^+^ mesenchymal-like cells to be a major cellular origin of renal fibrosis. In contrast, LeBleu et al. (182) questioned the specific contribution of pericytes to the myofibroblast population in the UUO model. Using genetic pericyte tagging approaches, they observed that most pericytes did not express αSMA. Moreover, they showed that specific deletion of pericytes neither changed the recruitment of myofibroblasts nor improved UUO-induced fibrosis. Based on these studies, LeBleu and coworkers concluded that pericytes have a negligible role as a source of myofibroblasts. Thus, additional studies are essential before the controversial role of pericytes as myofibroblast precursors is resolved.

**Figure 7.**
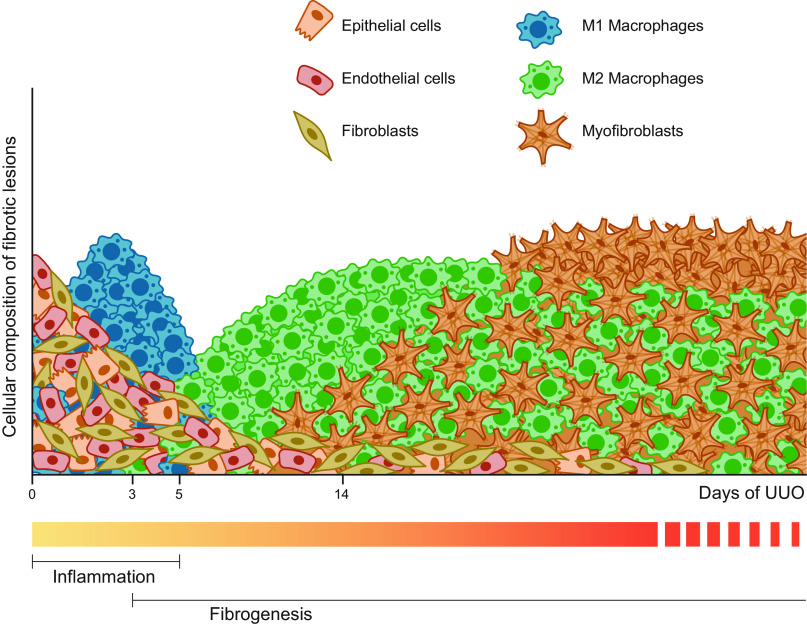
Time lapse of the cellular composition of the kidney during unilateral ureteral obstruction (UUO)-induced fibrogenesis. Before injury, the kidney mainly consists of fibroblasts, epithelial cells, and endothelial cells. Directly after UUO, the number of healthy cells starts to decline and there is a marked influx of proinflammatory M1 macrophages. Upon persistent injury, the resulting fibrotic lesions mainly consist of collagen-producing myofibroblasts and profibrotic M2 macrophages.

In conclusion, the current literature strongly supports the notion that myofibroblasts arise from a variety of cellular sources. Based on the latest research, ∼50% of myofibroblasts are supposed to be derived from resident fibroblasts, 35% from bone marrow-derived cells and macrophages, 5% from epithelial cells, and 10% from endothelial cells. However, these numbers need to be interpreted with caution. To date, resident fibroblasts and hematopoietic cells migrating into the renal tissue are considered the most important progenitors of myofibroblasts, and it is clear that the cellular composition of the kidney markedly changes during renal fibrosis (FIGURE 7). More insights can be obtained with the UUO model, and the implementation of advanced genetic analysis techniques, such as spatial transcriptomics, will hopefully provide an even better understanding of the origins of myofibroblasts in renal fibrosis.

### 7.4. The Distal Nephron—a Lesser-Known Player in TIF

The UUO model has primarily focused on the role of the proximal tubule in TIF and on late time points when the interstitial and fibrotic responses predominate (31, 32). However, the distal nephron, including the collecting duct, does significantly contribute to and modulate the pathophysiology and progression of renal injury following UUO. Hiatt and coworkers (222) investigated time points ranging from 1 to 14 days of UUO and demonstrated that obstruction of the urinary tract caused a two- to threefold increase in tubular dilation as well as a sixfold increase in accumulation of αSMA-positive myofibroblasts in the vicinity of the distal nephron. Myofibroblast accumulation was reported to occur rapidly in both the outer medullary region and, to a smaller extent, the cortical region. They also observed that the structural and cellular composition of the collecting duct was changed in response to obstruction. The number of AQP2-expressing principal cells and V-type proton ATPase (vATPase)-positive intercalated cells decreased by 65% and 75%, respectively, along with the disruption of E-cadherin localization. Notably, these features are also found in the distal and connecting tubules, indicating that the distal nephron is a major target of UUO-induced injury.

Several studies have demonstrated that collecting duct principal cells play a pivotal role in the development of tubulointerstitial fibrosis (223–225). Using a fetal monkey model in which UUO was performed at 70 days of gestation, Butt and coworkers (223) demonstrated that an abundance of collecting duct (CD) cells coexpress the intercalated cell marker carbonic anhydrase II and αSMA and these cells migrate through the basement membrane, suggesting a CD EMT, and thereby contribute to the development of progressive tubulointerstitial fibrosis. In other studies, tubulointerstitial inflammation and fibrosis were induced by collecting duct-specific disruption of β1-integrin (226) as well as of the E3 ubiquitin ligase Mib1, which is required for Notch signaling (227). In a recent study, specific inactivation of histone H3 K79 methyltransferase Dot1 in connecting tubules and collecting ducts facilitated the development of kidney fibrosis by increasing endothelin-1 levels in 14-day UUO mice (228). Moreover, it was revealed that kidney fibrosis after UUO is epigenetically regulated by Dot1 action in the connecting tubule and collecting ducts.

Taken together, the distal nephron contributes markedly to UUO-induced injury, indicating that the pathophysiology of obstructive nephropathy is complex and likely involves the entire nephron. This suggests that nephron segment-specific fibrotic factors might need to be taken into consideration in the preparation of targeted therapies.

## 8. MAJOR FACTORS DRIVING UUO-INDUCED DAMAGE AND POTENTIAL THERAPEUTICS

**Figure 8.**
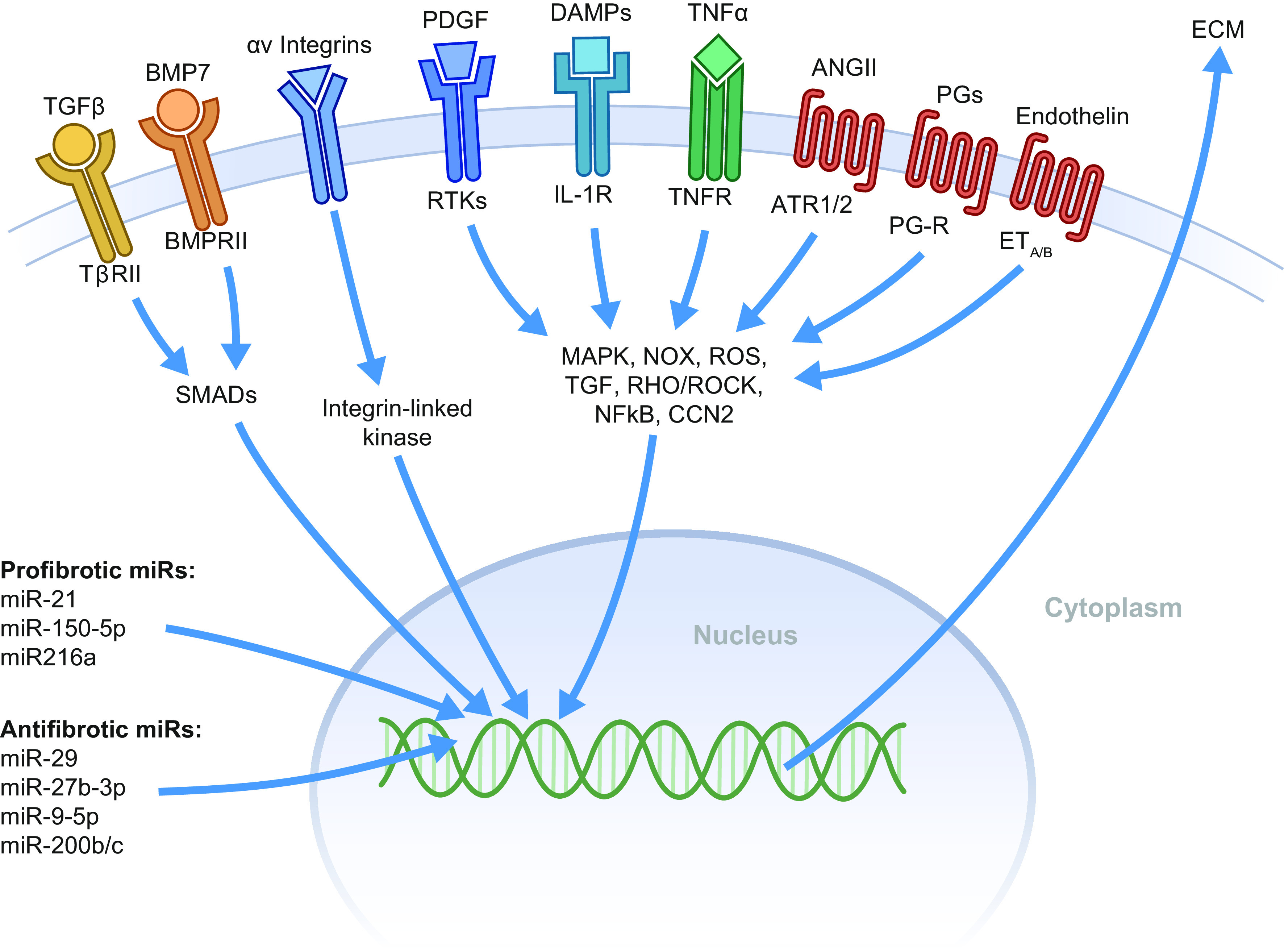
Schematic overview of the main signaling pathways involved in unilateral ureteral obstruction (UUO)-induced fibrogenesis. Binding of the various factors to their specific receptors mostly activates profibrotic signaling pathways, resulting in the formation and deposition of extracellular matrix (ECM) proteins; however, bone morphogenetic protein 7 (BMP7) is regarded as a protein that counteracts the profibrotic effects of transforming growth factor beta (TGFβ). In addition, prostaglandins (PGs) can be either pro- or antifibrotic depending on which receptor is activated. ANG II, angiotensin II; ATR1/2, angiotensin II receptor type 1 and type 2; BMPRII, bone morphogenetic protein receptor type II; CCN2, cellular communication network factor 2; DAMPs, damage-associated molecular patterns; IL-1R, interleukin-1 receptor; MAPK, mitogen-activated protein kinase; miR, microRNA; NFκB, nuclear factor kappa-light-chain-enhancer of activated B cells; NOX, nicotinamide adenine dinucleotide phosphate oxidases; PDGF, platelet-derived growth factor; PG-R, prostaglandin receptors; RHO/ROCK, Rho/Rho-associated protein kinase; ROS, reactive oxygen species; RTKs, receptor tyrosine kinases; TNFR, tumor necrosis factor alpha receptor; TNFα, tumor necrosis factor alpha; TβRII, transforming growth factor beta receptor II.

**Figure 9.**
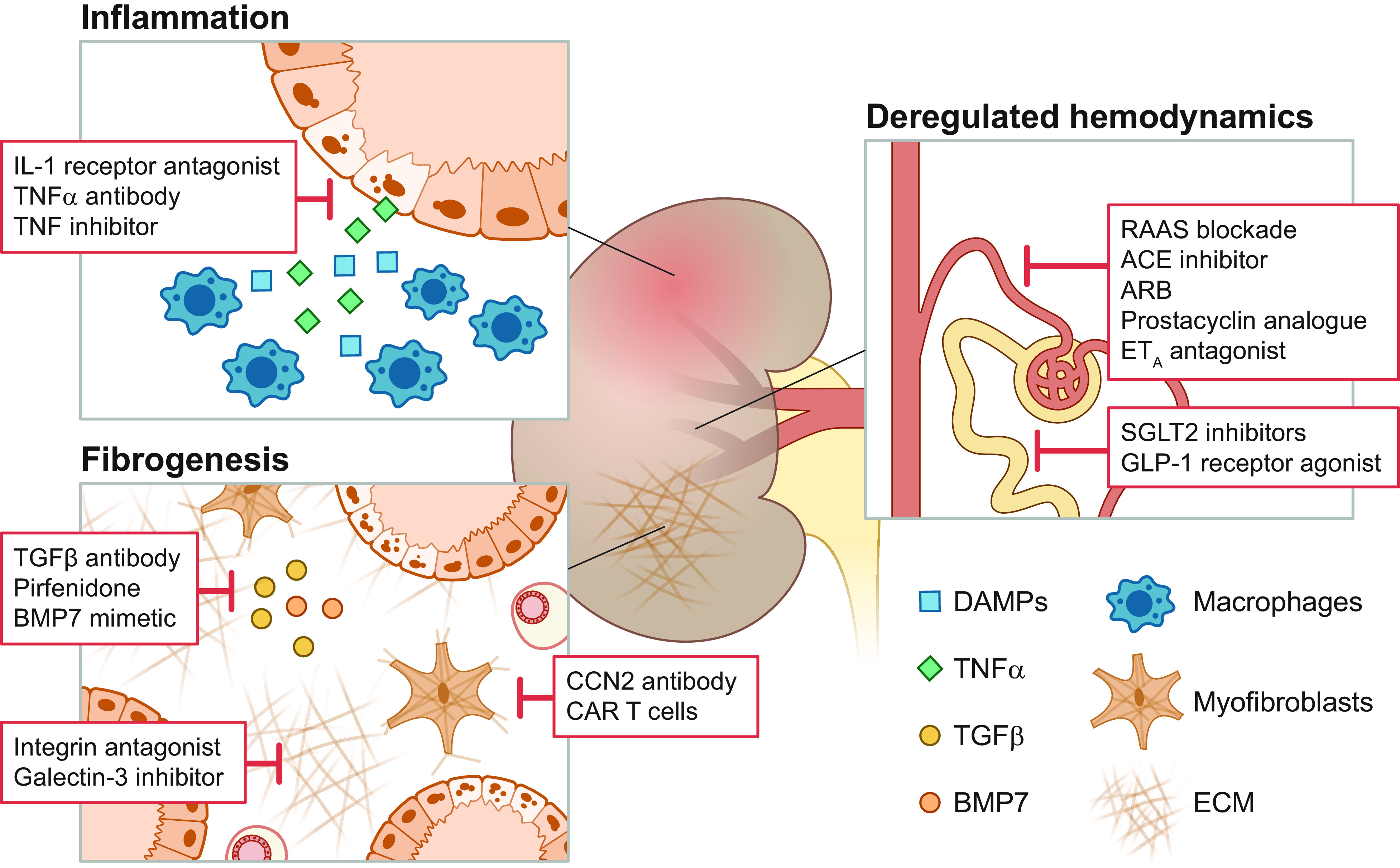
Overview of potential therapeutic targets for chronic kidney disease based on experimental unilateral ureteral obstruction (UUO) studies. In general, anti-inflammatory therapies are targeted against key cytokines such as interleukin 1 (IL-1) and tumor necrosis factor (TNF)α. Vascular targets are mainly associated with reducing hypertension-induced renal injury. Renal fibrosis can be mitigated by inhibition of the transforming growth factor beta (TGFβ) pathway, hampering integrin signaling, or reducing the number of collagen-producing myofibroblasts. ACE, angiotensin-converting enzyme; ARB, angiotensin receptor blockers; BMP7, bone morphogenetic protein 7; CAR T cells, chimeric antigen receptor T cells; CCN2, cellular communication network factor 2; DAMPs, damage-associated molecular patterns; ECM, extracellular matrix; ET_A_, endothelin receptor type A; GLP-1, glucagon-like peptide 1; RAAS, renin-angiotensin-aldosterone system; SGLT2, sodium-glucose cotransporter 2.

The molecular processes driving kidney fibrosis are complex and have far-reaching implications for the impairment of kidney function. In this section, we provide an overview of the most important signaling pathways and interactions related to UUO-induced fibrosis (summarized in FIGURE 8). Moreover, in FIGURE 9 we showcase potential therapeutic targets for preventing CKD progression supported by knowledge obtained from experimental UUO studies and delineate, based on these targets, which drugs are currently in the drug development pipeline or are already approved for the treatment of CKD (FIGURE 11). Details of the clinical trials are provided in TABLE 1.

**Table 1.** Characteristics of clinical trials pertaining to selected chronic kidney disease targets

Target	Drug	Phase	Condition	Clinical Trial	Status	Outcome	Ref.
TGF-β	LY2382770	II	DKD	NCT01113801	Terminated	No change in serum creatinine from baseline to end of treatment	(229)
	Fresolimumab	II	FSG	NCT01665391	Completed	The study was underpowered and did not meet the primary or secondary end points: remission in urinary protein-to- creatinine ratio, reduction in proteinuria, change in GFR.	(230)
	Pirfenidone	II	CKD	NCT04258397	Recruiting (estimated study completion date: December 2024)		
αv Integrins	STX-100	II	CAD	NCT00878761	Withdrawn	Withdrawn	
	GCS-100	II	CKD	NCT01843790	Completed	Pending	
CCN2	FG-3019	II	DKD	NCT00913393	Terminated	Terminated early for business purposes	
IL-1	Anakinra	II/III	CKD*	NCT02578394	Completed	Pending	(231)
	Rilonacept	II/IV	CKD	NCT00897715; NCT01663103	Completed	Treatment reduced systemic inflammation.	(232)
TNFα	Adalimumab	II	FSG	NCT00814255	Completed	The study was underpowered and did not meet the primary end point: reduction in proteinuria and stable GFR.	(233)
	Etanercept	II	ESRD	NCT00293202	Terminated	Unable to recruit sufficient patients	(234)
Prostacyclin	TRK-100STP	II/III	CKD	NCT01090037	Completed	No significant effect on the renal composite end point: doubling of serum creatinine or occurrence of ESRD	(235)
Endothelin	Atrasentan	III	DKD	NCT01858532	Terminated	Increased risk of heart failure hospitalization	(236,237)
	Avosentan	III	DKD	NCT00120328	Terminated	Induced significant fluid overload and congestive heart failure	(238)
GLP-1	Semaglutide	III	CKD	NCT04865770	Recruiting (estimated study completion date: July 2024)		
	Dulaglutide	IV	DKD	NCT05218915	Recruiting (estimated study completion date: January 2025)		
	Exenatide	IV	DKD	NCT02690883	Completed	Significant reduction of albuminuria	(239)
SGLT2	Canagliflozin	IV	CKD	NCT05309785	Recruiting (estimated study completion date: February 2025)		
	Dapagliflozin	III	CKD	NCT03036150	Completed (stopped early because of efficacy)	Significant effect on the composite end point: decline in GFR, reaching ESRD or death from renal or cardiovascular causes	(240)
	Empagliflozin	III	CKD	NCT03594110	Completed (stopped early because of efficacy)	Significant effect on the primary end point: first occurrence of progression of kidney disease or death from cardiovascular causes	(241)

CAD, chronic allograft dysfunction; CCN2, cellular communication network factor 2; CKD, chronic kidney disease; DKD, diabetic kidney disease; ESRD, end-stage renal disease; FSG, focal segmental glomerulosclerosis; GFR, glomerular filtration rate; GLP-1, glucagon-like peptide 1; IL-1, interleukin-1; SGLT2, sodium-glucose cotransporter 2; TGFβ, transforming growth factor beta; TNFα, tumor necrosis factor alpha; *As treatment for acute gout attacks.

### 8.1. Roles of TGFβ and BMP-7 in Obstructive Nephropathy

The TGFβ cascade plays a major role in tubulointerstitial inflammatory and fibrotic responses to experimental UUO (31, 242). The expression of *Tgfb1* mRNA increases in several nephron segments after 1 day of UUO, followed by upregulation of the protein (243–247). TGFβ transcripts are more abundant in the injured tubular epithelium and, to a lesser degree, in the fraction of resident and infiltrative macrophages (245, 248). Moreover, urinary TGFβ levels are increased in patients with ureteropelvic junction obstruction (249–251), and changes in these levels over time are associated with similar changes in the grade of hydronephrosis (252, 253), indicating that urine TGFβ can be a useful noninvasive tool for diagnosis of upper urinary tract obstruction in children.

TGFβ is a potent stimulator of the synthesis of ECM proteins and plays a central role in transdifferentiation of myofibroblasts (254–256). Blocking TGFβ with inhibitors, neutralizing antibodies, antisense oligonucleotides, or synthetic oligodeoxynucleotides (41, 257–261) can attenuate renal fibrosis in UUO models. The TGFβ signaling cascade involves phosphorylation of Smad2 and Smad3, which serve as downstream effectors, primarily by modulating target gene expression (256, 262) (FIGURE 8). Smad2 has a probable renoprotective role (263), whereas Smad3 is verified to be pathogenic because its targeted deletion (264) and deacetylation (265), or specific inhibition of TGFβ/Smad3 signaling (266), prevents UUO-induced fibrosis, suggesting that Smad3 may be a therapeutic target for renal damage related to obstructive nephropathy.

Bone morphogenetic protein-7 (BMP-7), formerly called osteogenic protein-1/OP-1, is a member of the TGFβ superfamily and is regarded as a protein that counteracts the profibrotic effects of TGFβ (262, 267). Moreover, BMP-7 can increase ECM degradation as well as reduce ECM formation by inactivating matrix-producing cells (267). Under disease conditions, BMP-7 expression is significantly downregulated, as detected in obstructive nephropathy (268, 269), and introduction of exogenous recombinant BMP-7 or reactivation of endogenous BMP-7 signaling pathways protects against UUO-induced fibrosis (47, 268, 270–273).

The TGFβ superfamily involves multiple signaling cascades and can interact with numerous other cell pathways, the details of which are too complex to review here and have been reviewed in detail previously (31, 256, 262). Taken together, the regulation of TGFβ-induced renal fibrosis is complex.

#### 8.1.1. TGFβ as therapeutic target in CKD.

Substantial preclinical as well as clinical evidence indicates that TGFβ plays a crucial role in promoting renal fibrosis, suggesting TGFβ to be a major therapeutic target in CKD patients (FIGURE 9). However, because TGFβ regulates many biological functions in normal human physiology as well as in pathological conditions, developing successful therapies that interrupt the TGFβ pathway has been very challenging. Global blockade of TGFβ can have deleterious effects, especially in CKD, where long-term treatment is required (274). Nevertheless, several clinical trials have been conducted to evaluate the effect of pharmacological blockade of TGFβ in fibrotic kidney disease with variable success. A multicenter phase II clinical trial using a humanized neutralizing antibody against TGFβ (LY2382770) in diabetic kidney disease showed no clinical benefits (229). A subsequent phase II multicenter, double-blind, placebo-controlled randomized study of another neutralizing antibody (fresolimumab) against all three TGFβ isoforms was evaluated in patients with primary focal segmental glomerulosclerosis (FSGS). Although fresolimumab was well tolerated, no statistical difference in estimated GFR (eGFR) was observed between the groups (230). A single-center pilot study showed that oral administration of pirfenidone, a small-molecule compound inhibiting TGFβ synthesis, significantly slowed the decline in renal function in patients with FSGS (275). Moreover, another phase I/II randomized placebo-controlled trial in patients with established diabetic nephropathy showed promising results for pirfenidone in improving kidney function, but without improving proteinuria (276). Interestingly, another phase II randomized, double-blind, placebo-controlled study on pirfenidone is planned for patients with CKD to evaluate whether pirfenidone can prevent CKD progression. This study will be completed in December 2024 (ClinicalTrials.gov: NCT04258397). However, despite promising results in preclinical studies, therapeutic interventions targeting TGFβ in humans have been disappointing. Further research is needed on the pathways that trigger TGFβ-induced fibrosis to identify alternative therapeutic strategies for mitigating kidney fibrosis in CKD.

### 8.2. Role of Integrins in Obstructive Nephropathy

Integrins are a large family of transmembrane cell adhesion and signaling receptors, consisting of α- and β-subunits that are able to connect the cytoskeleton with the ECM (FIGURE 8). In mammals, 18 α- and 8 β-subunits have been identified, forming a total of 24 different integrin heterodimers (277). Several studies have demonstrated that integrins, specifically αv (alpha-vee)-containing integrins, play an important role in controlling the activation of TGFβ. Myofibroblasts express the αv-containing integrins (αvβ1, αvβ3, αvβ5, αvβ6, αvβ8), which can recognize the amino acid sequence Arg-Gly-Asp (RGD), allowing them to bind to and activate latent TGFβ (reviewed in Refs. 278, 279). In line with this, Ma and coworkers (280) showed that disruption of αvβ6-mediated TGFβ activation could protect against tubulointerstitial fibrosis in mice after 14-day UUO. Likewise, inhibition of integrin αvβ1 or integrin β3 ameliorated UUO-induced fibrosis (281, 282). Moreover, in a recent study, an integrin αvβ3-based drug delivery system that targets myofibroblasts was constructed and it was shown that specific targeting of αvβ3-positive myofibroblasts alleviated UUO-induced fibrosis (283).

Integrin-linked kinase (ILK) also plays a role in the development of kidney fibrosis. ILK is an intracellular serine/threonine protein kinase that interacts with the cytoplasmic domains of the β-integrin subunit, resulting in integrin-mediated signaling (284). ILK expression is increased in response to UUO in a time-dependent manner (285), and its overexpression aggravates renal fibrosis (286). Li and coworkers (287) demonstrated that inhibition of ILK activity attenuates ECM production and myofibroblast activation in UUO-induced fibrosis.

Overall, these studies indicate that the integrin signaling pathways play a critical role in the development and progression of kidney fibrosis and might represent novel targets for antifibrotic therapies (FIGURE 9). However, the complexity of integrin biology, with multiple integrin subtypes involved in kidney fibrosis, suggests that a small-molecule integrin modulator targeting several integrin subtypes might be an ideal antifibrotic therapy. Interestingly, Zhou and coworkers (288) demonstrated that MK-0429, an equipotent pan-inhibitor of multiple αv integrins, reduces proteinuria, kidney fibrosis, and collagen accumulation in a rat model of diabetic nephropathy. This pan-αv inhibitor has recently been patented by Merck for CKD treatment (289).

### 8.3. Role of Growth Factors in Obstructive Nephropathy

#### 8.3.1. Platelet-derived growth factor.

The platelet-derived growth factor (PDGF) family consists of five dimeric glycoproteins (PDGF-AA, -AB, -BB, -CC, and -DD), which signal via either receptor PDGFRα or PDGFRβ (FIGURE 8). These two receptors are expressed by mesenchymal cells in all organs, and their activation drives proliferation, migration, and production of ECM, reflecting a key role of PDGF in numerous (patho)physiological processes, including fibrosis of the lung, kidney, liver, and heart (290–292). With regard to UUO-induced renal fibrosis, Sommer and colleagues (293) used Northern blot analysis to demonstrate that *Pdgfb* mRNA levels slowly increased after obstruction and reached the highest levels on days 10 and 15. In contrast, expression of *Pdgfrb* remained elevated for 25 days (293). Taneda and coworkers (294) used immunohistochemistry to evaluate the expression profile of PDGF ligands and receptors in UUO kidneys. They observed in mice that PDGF-A was expressed in vascular smooth muscle cells and some infiltrating cells in UUO kidneys but not in cortical interstitial cells. On day 4 after UUO, PDGF-B was observed in proximal and distal tubules as well as in interstitial cells; in the latter, PDGF-B was still present on day 14. In healthy kidneys, PDGF-C was observed in various endothelial cells, and its expression profile did not change after UUO; however, in another study PDGF-C was highly expressed in infiltrating macrophages in UUO kidneys (193). PDGF-D was present in interstitial cells on day 4 after UUO, especially around injured tubules, and expression levels were even higher on day 14. In another study on fibrotic kidneys, dilated tubules coexpressed PDGF-D and AQP1, indicating that proximal tubules and/or descending limbs of Henle’s loop can express PDGF-D de novo upon injury (295). Furthermore, PDGF-D is dispensable for normal renal development and physiological function and may represent an attractive therapeutic target in CKD.

In UUO mice, PDGF receptors PDGFRα and PDGFRβ are mainly expressed in interstitial cells from day 4 (294). Chen and colleagues (296) reported a time-dependent increase in PDGF isoforms and its receptors after UUO as well as in the phosphorylation of PDGFRα and PDGFRβ. Moreover, using collagen 1-green fluorescent protein reporter mice, they demonstrated that PDGFRα and PDGFRβ were mainly expressed in pericytes and interstitial myofibroblasts in both healthy and UUO kidneys (296). Despite the clear role of PDGF signaling in fibrogenesis, it has been difficult to utilize this pathway as a therapeutic target, especially in clinical settings. Therefore, direct targeting of the PDGF pathway might not be the therapeutic way forward; however, the distinct, cell-specific expression profile of PDGF receptors opens other possibilities as described by Poosti and coworkers (297), who used PDGFRβ as a docking receptor for myofibroblast-specific delivery of IFNγ to mitigate UUO-induced fibrosis.

#### 8.3.2. Connective tissue growth factor/cellular communication network factor 2.

In 2018, the HUGO Gene Nomenclature Committee decided that the official name for connective tissue growth factor (CTGF) would become cellular communication network factor 2 (CCN2), partly based on the fact that the old name was misleading considering that the gene does not encode a growth factor (298). Historically, CCN2 was regarded as a growth factor and a proinflammatory cytokine; however, it is now clear that CCN2 is a matricellular protein involved in various cellular functions, including matrix remodeling (299) (FIGURE 8). The multiplicity of CCN2 functions owes to its complex protein structure. CCN2 consists of four functional domains, viz. module I, homologous to insulin-like growth factor binding protein; module II, a von Willebrand factor type-C repeat; module III, a thrombospondin type-1 repeat; and module IV, a cysteine knot-containing domain (CT), with a “hinge” region between the second and third domains (300). Based on its pericellular localization and complex structure, it is believed that CCN2 can regulate cell functions through interactions with diverse receptors and macromolecules in various extracellular and intracellular compartments. Moreover, the complete protein can be cleaved by proteases, such as MMP-2, resulting in the release of various biologically active fragments (301, 302). CCN2 expression is associated with a variety of fibrotic diseases, including renal fibrosis (303–305). CCN2 can stimulate fibrogenesis by promoting the recruitment of inflammatory cells, stimulating fibroblast proliferation and activation, and enhancing ECM production. These profibrotic effects are mediated by interaction with, among others, TGFβ, BMPs, MMPs, integrins (e.g., αvβ3, αvβ5), Wnt, and fibronectin (304, 305). In 1998, Ito and coworkers (306) reported that CCN2 was highly expressed in fibrotic lesions and at sites of chronic tubulointerstitial damage in patients with CKD of various origins. In 2004, the role of CCN2 in UUO-induced renal fibrosis was proven by Yokoi and colleagues (307). Using a CCN2 antisense oligonucleotide, they demonstrated that CCN2 blockade mitigated UUO-induced fibronectin and collagen 1 expression and deposition without impacting the proliferation of tubular and interstitial cells (307). In 2010, a phase 1 trial showed that treatment of patients with microalbuminuric diabetic kidney disease with a monoclonal antibody to CCN2 reduced albuminuria (308). Since then, a variety of therapeutics targeting CCN2 have been developed (300); however, unfortunately, no clinical trials are ongoing for CCN2 and CKD.

### 8.4. Role of Cytokines in Obstructive Nephropathy

#### 8.4.1. Damage-associated molecular patterns.

**Figure 10.**
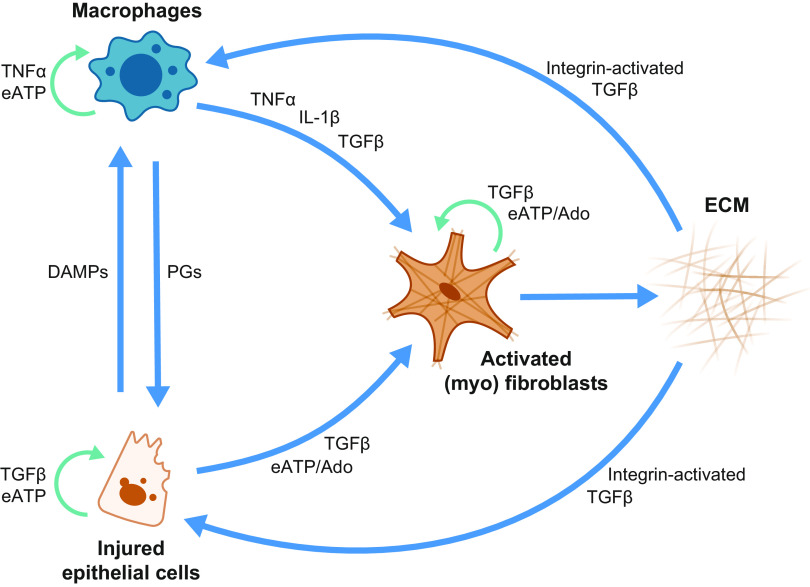
Simplified representation of a signaling network driving renal fibrosis. Factors related to the damage-associated molecular pattern (DAMP), prostanoid, purinergic, and transforming growth factor beta (TGFβ) signaling pathway can stimulate key cellular players in both an autocrine (green arrows) and a paracrine (blue arrows) fashion, thereby creating an autoinductive feedforward loop resulting in perpetual activation of the profibrotic machinery. For instance, autocrine extracellular ATP (eATP) signaling promotes inflammasome activation in macrophages, resulting in the production of interleukin-1 beta (IL-1β), which, in a paracrine manner, activates fibroblasts. As a consequence, fibroblasts increase the expression of integrins that are capable of activating latent, extracellular matrix (ECM)-bound TGFβ. Subsequently, autocrine TGFβ signaling maintains fibroblast activation, and paracrine TGFβ signaling promotes the release of cytokines by epithelial cells resulting in macrophage recruitment. Ado, adenosine; PGs, prostaglandins; TNFα, tumor necrosis factor alpha.

UUO causes sterile inflammation, which is an inflammatory response to physical, chemical, or metabolic noxious stimuli, in the absence of pathogens, characterized by the release of damage-associated molecular patterns (DAMPs) (309). Several DAMPs, including high-mobility group box 1, IL-1α, IL-33, soluble uric acid, extracellular ATP (eATP), and S100A8/A9, are involved in UUO-induced fibrosis (310–314). These DAMPs interact with various receptors, such as toll-like receptors, the IL-1 receptor family, and P2 purinergic receptors (314–316), and receptor binding results in inflammasome assembly, caspase activation, and maturation of IL-1β and IL-18 and promotes fibrogenesis (314, 317, 318) (FIGURE 8 AND FIGURE 10). Considering the central role of the IL-1 family of cytokines in renal fibrosis, they are potentially interesting therapeutic targets (FIGURE 9). Several clinical trials have been conducted in CKD patients using anakinra, a nonglycosylated form of the naturally occurring human IL-1 receptor antagonist (IL-1Ra). At the time of writing, one trial (NCT04844814) was about to start, one was withdrawn because of failure in enrolling patients (NCT03062176), and four others were completed [NCT03141983, NCT02578394 (231), NCT02278562, and NCT00420290]. The completed trials showed that administration of anakinra to maintenance hemodialysis patients for 4 wk successfully controlled the inflammatory response, as evidenced by a significant reduction in C-reactive protein and IL-6 levels (319). In another two-site, double-blind trial (NCT01663103 and NCT00897715), the effect of an IL-1 trap, rilonacept, was tested in CKD patients, and it was found to reduce systemic inflammation (232). Recently, canakinumab, a human monoclonal antibody against IL-1β, was shown to mitigate EMT of HK-2 cells (320). However, the efficacy of other (in)direct cytokine inhibitors, such as emricasan, serelaxin, and salidroside, in the treatment of CKD needs to be further evaluated (317). Of note, another cytokine that might be a promising therapeutic target is IL-11 (321). With regard to eATP-mediated purinergic signaling, it has been demonstrated that the P2X_7_ receptor is involved in UUO-induced inflammation and fibrosis (322), and treatment of UUO rats with brilliant blue G, a P2X_7_ receptor antagonist, reduced renal inflammation, collagen synthesis, and renal cell apoptosis (323). Noteworthy, several P2X_7_ receptor inhibitors are already being tested in clinical trials for the treatment of inflammatory diseases (324); however, whether these compounds can be used for the treatment of CKD still needs to be assessed.

#### 8.4.2. Adenosine.

Extracellular adenosine is an endogenous signaling molecule that regulates inflammatory and fibrotic processes in various organs (325–327). Under normal, normoxic conditions, adenosine is present in low concentrations in the extracellular space of the kidney. However, after hypoxia or renal injury, the levels of extracellular adenosine rise significantly, in part due to cell death-mediated release of ATP into the pericellular space (328). Extracellular adenosine can signal through four different G protein-coupled P1 receptors (A_1_, A_2A_, A_2B_, and A_3_) that either stimulate (A_2A_ and A_2B_) or inhibit (A_1_ and A_3_) adenylyl cyclase activity and cAMP production in the cell (328, 329). The various receptor subtypes are differentially expressed depending on the cell type: A_2A_ is the predominant receptor subtype on immune cells (330), whereas A_2B_ is the predominant adenosine receptor expressed on isolated renal fibroblasts (327). As a consequence, the receptors also contribute differently to renal fibrogenesis. Using the UUO model, several studies demonstrated that deletion of the A_2A_ receptor increases renal fibrosis (331, 332) whereas activation of the receptor slows down the fibrotic process; however, treatment did not fully protect against UUO-induced injury (331, 333). Conversely, it has been demonstrated that deletion of the A_2B_ receptor attenuates UUO-induced fibrosis (334). Taken together, these studies reveal that the adenosine receptor subtypes should be modulated differently in order to achieve therapeutic efficacy. Along these lines, Pak et al. (335) recently demonstrated that LJ-4459, a newly synthesized dual-acting ligand that acts as an A_2A_ agonist and an A_3_ antagonist, reduced fibrosis in 6-day UUO mice.

#### 8.4.3. NLRP3 inflammasome.

Inflammasomes, first described in 2002 by Martinon and colleagues (336), are intracellular multiprotein complexes, mainly consisting of a pattern recognition receptor, an apoptosis-associated speck-like protein, and pro-caspase-1, that are known to play an important role in organ fibrosis (337). Classical activation of the inflammasomes, for instance by DAMPs, results in caspase-1-mediated maturation of IL-1β, IL-18, and IL-33, thereby promoting inflammation and fibrogenesis (337). The NLRP3 inflammasome is currently the most studied and characterized inflammasome and well known to contribute to CKD (337–339). In 2010, Vilaysane et al. (340) reported that NLRP3 gene and protein expression increased progressively over a 14-day time course in UUO mice. During that time, they also observed increased levels of activated caspase-1 and mature IL-1β and IL-18 (340). Moreover, they demonstrated that NLRP3 deletion attenuated tubular injury, tubulointerstitial inflammation, and fibrosis following UUO (340). These findings were corroborated by Guo and coworkers (341), who demonstrated that NLRP3 deficiency ameliorates mitochondrial dysfunction and alleviates renal fibrosis in 14-day UUO mice. Conversely, Pulskens and colleagues (342) reported in mice that after 1-day UUO NLRP3 preserved renal integrity and provided protection against early tubular injury and interstitial edema. Despite these conflicting results, the inflammasome–IL-1/IL-18 axis appears to a promising therapeutic target for CKD. Recently, it was demonstrated that gemigliptin, a dipeptidyl peptidase-4 inhibitor, mitigated UUO-induced tubular atrophy and renal fibrosis in mice by downregulating NLRP3 activity (343). As described previously, several drugs that target cytokines downstream of NLRP3 activation, e.g., anakinra and rilonacept, are currently being evaluated for the treatment of CKD; however, selective NLRP3 inhibitors, such as CY-09 and OLT1177 (dapansutrile) (344, 345), are still only undergoing preclinical testing (346, 347).

#### 8.4.4. Tumor necrosis factor-α.

Tumor necrosis factor-α (TNFα) is produced by various cells including macrophages, glomerular mesangial cells, and tubular epithelial cells (348). It acts via two receptors, TNF receptor (TNFR)1 and 2, the activation of which results in the expression of a variety of transcription factors, cytokines (e.g., IL-1β and CCL2), growth factors (e.g., TGFβ), receptors, cell adhesion molecules (e.g., ICAM-1 and VCAM-1), mediators of inflammatory processes, acute phase proteins, and major histocompatibility complex proteins (291, 348) (FIGURE 8). Notably, TNFRs can be shed, and soluble TNFR can mitigate inflammation and fibrosis by neutralizing free TNFα (291). More than 25 years ago, Kaneto et al. (349) demonstrated significant increase in the expression of *Tnf* mRNA in obstructed kidneys at 1 (×2), 2 (×2.7), 4 (×3.6), 24 (×2.7), 72 (×1.8), and 120 (×2.8) h after UUO compared with that in the contralateral kidney of the same rats. In 1999, Guo and colleagues (350) demonstrated that TNFα and its two receptors are involved in UUO-induced fibrosis. Using knockout (KO) mice, they demonstrated that deletion of TNFR1 markedly reduced the interstitial volume of the 5-day UUO kidney from 33% [wild-type (WT) mice] to 19.4%, whereas that of TNFR2 KO mice was significantly decreased to 25.4% (350). Moreover, they observed a reduction in collagen IV and αSMA matrix scores and reduced nuclear factor kappa-light-chain-enhancer of activated B cells (NFκB) activation (350). They postulated that TNFα and ANG II together accounted for ∼70–80% of the pathophysiological changes in obstructive nephropathy (351). Inhibition of TNFα with infliximab, etanercept, or PEG-sTNFR1, a pegylated form of soluble TNFR1, decreased albuminuria and slowed down CKD progression in animal models (352–354); however, no clinical studies have been conducted. The FONT phase II clinical trial (NCT00814255) was designed to assess the efficacy of adalimumab in patients with resistant focal segmental glomerulosclerosis. Unfortunately, recruitment fell short of enrollment goals (233). In a pilot study to evaluate the efficacy of etanercept in chronic hemodialysis patients (NCT00293202), no changes in inflammation markers were observed (234).

### 8.5. Role of Vasoactive Peptides in Obstructive Nephropathy

#### 8.5.1. Angiotensin II.

As described above, RAS and ANG II have been known for decades to play an important role in the pathogenesis of UO (78, 79). In UUO kidneys, activation of RAS is related to mechanical stretching, leading to inflammation, oxidative stress, and fibrosis (355, 356). ANG II, via AT(1) and AT(2) receptors, activates the NFκB signaling pathways, contributing to increased production of TNFα, IL-6, and MCP-1, which promotes inflammation, apoptosis, and oxidative stress (FIGURE 8). Consequently, blockade of the ANG II system, for example, with ACE inhibitors, AT1 receptor antagonists, and thymoquinone, reduces the expression of NFκB-related proinflammatory genes in UUO kidneys, thereby improving inflammation and oxidative damage (357–360). The interaction between ANG II and TNFα was studied in further details by using a genetic-pharmacological approach with a combination of AT1- and TNFα receptor- (TNFR1 and TNFR2) KO mice and pharmacological manipulation (350, 351). Guo and coworkers subjected double TNFα receptor KO mice to 5-day UUO and then treated them with the ACE inhibitor enalapril to assess the contribution of both systems to the development of renal fibrosis (351). Treatment with enalapril further decreased fibrosis progression in the double TNFα receptor KO mice after 5-day UUO, suggesting an interaction between ANG II and TNFα systems. Moreover, ANG II activates TGFβ signaling, leading to increased production of ECM as well as fibrosis; specific ANG II receptor antagonists or ACE inhibition was reported to ameliorate the UUO-induced fibrosis (361–363). In line with these studies, it has been shown that KO or transgenic mice for the AT1 receptor or angiotensinogen gene develop less TIF than WT mice during UUO (364, 365). In contrast, Nishida and coworkers (366) demonstrated that stimulation of the AT1 receptor can also be beneficial in 14-day UUO. They performed bone marrow transplantation from AT1 receptor KO mice to WT mice and showed that kidneys from mice with AT1-deficient bone marrow display more fibrotic changes and fewer infiltrating macrophages than those from nontransplanted WT mice after 14 days of UUO. Because infiltrating macrophages are assumed to play a critical role in the evolution of renal fibrosis, this study suggests that the AT1 receptors on bone marrow-derived macrophages might promote preservation of the renal parenchymal architecture during development of renal fibrosis in response to UUO.

Activation of RAS and the increase in ANG II levels leads to overproduction of reactive oxygen species (ROS) in UUO kidneys (355). The mitochondria play a central role in controlling ROS production, and in UUO mitochondrial dysfunction is associated with increased generation of ROS leading to oxidative stress. In addition to mitochondria, nicotinamide adenine dinucleotide phosphate (NADPH) oxidases (NOXs) and endoplasmic reticulum (ER) are important sources of ROS that can activate different signaling pathways, thereby contributing to inflammation and fibrosis (367). ANG II is able to stimulate NOX-derived ROS production via binding to AT1 receptors (368), and in UUO models NOX2 and NOX4 are upregulated after 7 days of UUO (369, 370). Treatment with an ANG II receptor blocker, fimasartan, attenuated the UUO-induced expression of NOX2 and NOX4 but enhanced a nuclear factor erythroid 2-related factor 2 (Nrf2)-mediated antioxidant defense response, which is critical for neutralizing ROS and reducing oxidative stress as well as inflammation and fibrosis (369). Furthermore, in 14-day UUO mice, ANG II upregulation of NOX2 could be attenuated by β-aminoisobutyric acid (BAIBA), a natural thymine catabolite, leading to decreased oxidative stress and fibrosis (371). In line with this, Cheng and coworkers (372) showed that treatment with apocynin, a NOX2/NOX4 inhibitor, reduced oxidative stress and myofibroblast accumulation in 7-day UUO rats. Likewise, ANG II activation of the AT1 receptor increased the levels of NOX subunits, including p22/p47/p67, in 7-day UUO mice, resulting in ROS overproduction, fibrosis, and loss of kidney function (373). Thus, these studies indicate that increased production of ANG II by RAS activation is associated with oxidative stress, promoting renal damage and inflammation, which leads to kidney fibrosis.

It has been proposed that the intrarenal RAS is as important as systemic RAS in terms of renal pathophysiology, including in UO (374–376). Recently, Figueroa and colleagues (374) demonstrated that the intrarenal RAS is activated in the cortex of UUO kidneys as reflected by increased renin expression. Moreover, it has been shown that suppression of intrarenal RAS by angiotensinogen (AGT) antisense RNA prevents UUO-induced TGFβ, fibronectin, and collagen type I expression (377). Together, this suggests that targeting the intrarenal RAS may have significant clinical implications for the treatment of renal fibrosis.

#### 8.5.2. Renin-angiotensin system as a therapeutic target in CKD.

**Figure 11.**
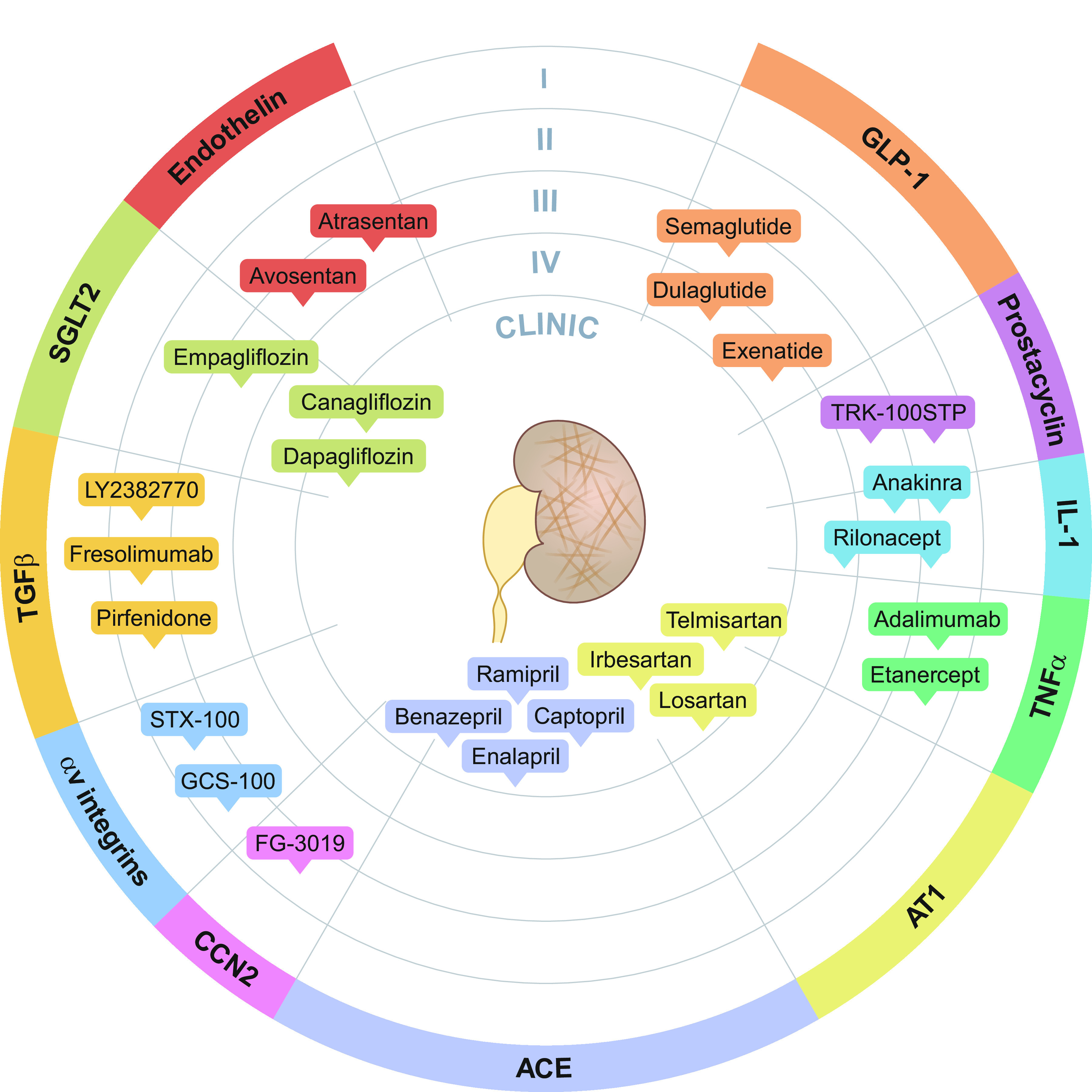
Overview of the current drug development pipeline for chronic kidney disease containing drugs that target pathways that have been shown to prevent renal injury in experimental models of unilateral ureteral obstruction (UUO). ACE, angiotensin-converting enzyme; AT1, angiotensin II receptor type 1; CCN2, cellular communication network factor 2; GLP-1, glucagon-like peptide 1; IL-1, interleukin 1; SGLT2, sodium-glucose cotransporter 2; TGFβ, transforming growth factor beta; TNFα, tumor necrosis factor alpha.

Current therapeutic strategies to delay CKD progression focus primarily on treating the underlying conditions, such as high blood pressure and glucose levels. Clinically, ACE inhibitors and ANG II receptor blockers are therefore cornerstone therapeutics to reduce hypertension, proteinuria, progression of CKD, and cardiovascular risk (378, 379) (FIGURE 9 AND FIGURE 11). Several clinical studies initiated in the late 1980s have shown that blockers of RAS activity can improve kidney function in patients with progressive CKD over the long term (379). In most of these studies, the primary end points were defined as the occurrence of ESRD, doubling of serum creatinine levels, or a combination of both. Moreover, a recent double-blind randomized placebo-controlled trial demonstrated that losartan administration contributes to renal function recovery, as shown by renographic GFR improvement, in anuric and oliguric patients with a solitary obstructed kidney (17). However, the promising preclinical effect demonstrating that RAS blockade can reduce renal inflammation and fibrosis remains to be demonstrated clinically.

#### 8.5.3. The prostaglandin system.

The prostaglandin system is activated in response to obstructive nephropathy. Numerous studies have shown that obstruction of the urinary tract is associated with a marked induction of COX-2, as well as an increase in prostaglandin and thromboxane synthesis (78, 103, 104, 108, 116–118, 380, 381). COX-2 distributes differentially in various regions of the kidneys upon UUO. Compared with the contralateral nonobstructed kidney, cortical COX-2 expression is reduced in the obstructed kidney but is markedly induced in both the outer and inner medulla, indicating that COX-2 may contribute to distinct regional hemodynamic alterations in response to UUO. Conversely, COX-1 expression is identical in the obstructed and contralateral nonobstructed kidneys, highlighting COX-2 as the main player in obstructive nephropathy (116).

There is some controversy regarding the role of COX-2 in renal injury secondary to UO. Inhibition of COX-2 prevented UUO-induced apoptosis and fibrosis (382, 383), indicating that COX-2 has detrimental effects. In line with these findings, we inhibited COX-2 using target-specific gene silencing via RNA interference (RNAi). We designed chitosan/COX-2 siRNA nanoparticles that silenced COX-2 specifically in the macrophages. These nanoparticles were administered via intraperitoneal injection, and the character of these chitosan nanoparticles and the macrophage-rich milieu in the peritoneum aided their proficient uptake by the macrophages (384). These macrophages were then subsequently recruited to the obstructed kidney; in 3-day UUO mice with macrophage-specific knockdown of COX-2, renal inflammation, apoptosis, and fibrosis were diminished (385, 386). Thus, COX-2 inhibition using RNAi may represent a potential therapeutic approach.

Contrary to these findings, Kamata et al. (387) showed that meloxicam, a selective COX-2 inhibitor, aggravated 5-day UUO-induced fibrosis, indicating a protective effect of COX-2-derived prostaglandins against UUO-induced renal damage. Consistent with these findings, we demonstrated higher levels of apoptosis as well as tubular damage in COX-2 KO mice subjected to 3 and 7 days of UUO (388), supporting the protective role of COX-2. The microsomal PGE2 synthase, which is responsible for the production of PGE2, has likewise been shown to exert a protective effect against inflammation and renal fibrosis in 7-day UUO mice (389). This was further supported by studies focusing on the PGE_2_, EP_2_, and EP_4_ receptors (390). PGE_2_ can bind and activate four different EP receptors, EP_1–4_. We and others have demonstrated increased expression of EP_2_ and EP_4_ receptors in response to 7-day UUO (388, 389, 391). Moreover, we recently demonstrated that stimulation of the EP_2_ receptor mitigates renal fibrogenesis in 7-day UUO mice and human precision-cut kidney slices (PCKS) (392). PCKS is a unique translation ex vivo model of renal fibrosis that is ideal for studying multicellular processes because cellular diversity and organ architecture are maintained in the slices. The slices are prepared from functional and macroscopically healthy cortical tissue obtained from tumor nephrectomies (393). In addition, Nakagawa and coworkers (394) showed aggravated renal injury in mice lacking the EP_4_ receptor in response to 7-day UUO. Taken together, these studies confirm that the activation of EP_2_ and EP_4_ receptors might also protect against UUO-induced damage, indicating that these receptors may be involved in the cytoprotective effect of COX-2 activation during UUO (FIGURE 8). In contrast, we demonstrated that inhibition of the EP_1_ receptor diminished fibrosis in 7-day UUO mice and mitigated early- and late-stage renal fibrosis in human PCKS prepared from patients with established renal fibrosis (395), indicating that the EP_1_ receptor is also a promising target for preventing renal fibrosis.

Prostaglandin I_2_ (PGI_2_) or prostacyclin also plays an important role in UUO-induced damage. PGI_2_ is primarily synthesized in endothelial cells and has a strong vasodilation function. In this regard, it was recently demonstrated that a prostacyclin analog, beraprost sodium, could attenuate renal fibrosis by improving renal microcirculation and suppressing EndoMT progression in mice subjected to 7-day UUO (396). Moreover, ONO-1301, a PGI_2_ receptor (IP) agonist, mitigated TIF in 7-day UUO mice (397).

Prostaglandin D_2_ (PGD_2_) has likewise been demonstrated to affect the development of renal fibrosis. It can interact with two receptors, viz., the DP_1_ receptor and the DP_2_/CRTH2 receptor. In a study on renal fibrosis, the PGD_2_-CRTH2 pathway was found to be involved in the progression of inflammation and TIF (398). Ito and coworkers (399) blocked the activation of CRTH2, using an antagonist or genetic KO, and showed that UUO-induced TIF was ameliorated in CRTH2 KO mice or in mice treated with the CRTH2 antagonist, suggesting that the PGD_2_-CRTH2 pathway promotes renal fibrosis.

Taken together, these studies reveal a counterbalance between the intrarenal handling of COX-2, that is, the detrimental versus beneficial effects of COX-2 may depend on the cellular expression and localization of COX-2 in the obstructed kidney. This suggests that protective COX-2-mediated endogenous prostaglandin production may take place in resident cells in response to UUO. Hence, the COX-2/prostaglandin signaling pathway is complex and can be regulated at multiple steps. Among these steps, the COXs and prostaglandin synthases are of importance in the production of prostaglandins, which can then bind and activate specific cytoprotective receptors and subsequently reduce renal injury and fibrosis.

#### 8.5.4. The COX/prostaglandin system as a therapeutic target in CKD.

Nonsteroidal anti-inflammatory drugs (NSAIDs), which are nonspecific inhibitors of both COX-1 and COX-2 as well as selective COX-2 inhibitors, are among the most commonly used drugs for treatment of pain, fever, and inflammation. However, the use of NSAIDs is associated with an increased risk of renal and cardiovascular complications, such as compromised GFR, elevated blood pressure, and peripheral edema (400, 401). Gooch and coworkers (402) performed a prospective community-based study of >10,000 elderly patients to determine the association between NSAID use and CKD progression. They showed a linear relationship between a high cumulative NSAID exposure and an increased risk for CKD progression in an elderly community-based cohort. Moreover, Whelton and coworkers (403) reviewed >50 clinical studies involving >13,000 subjects to study the renal safety profile of the selective COX-2 inhibitor celecoxib. They reported that the overall incidence of renal adverse events was greater for celecoxib treatment compared with that for placebo but was similar to that for NSAIDs.

Considering the high adverse effects of NSAIDs on the kidney, an increasing number of preclinical studies are now focusing on evaluating the effect of prostaglandin analogs or receptor agonist/antagonists in CKD animal models. In the future, relevant clinical studies will be crucial to verify the effect of prostaglandin analogs or receptor agonist/antagonist in the treatment of renal fibrosis in CKD patients. A few clinical trials have been conducted. The CASSIOPEIR trial studied the effect of prostacyclin analog beraprost sodium (TRK-100STP) in patients with CKD (either primary glomerular disease or nephrosclerosis). This randomized, double-blind placebo-controlled study, including 892 CKD patients, has been conducted at 160 sites in seven Asia-Pacific countries and regions. Unfortunately, no beneficial effects of TRK-100STP in preventing CKD progression were observed over placebo (235).

#### 8.5.5. Endothelin.

Endothelins (ETs) are a family of three potent vasoactive peptides (ET-1, ET-2, and ET-3). ET-1 is the main isoform in the kidney and plays an important role in the regulation of renal function (404). ET-1 can bind two different receptors, namely ET_A_ and ET_B_ (FIGURE 8). Both receptor subtypes are present in the kidney but have different localization and functions. The ET_A_ receptor is mainly expressed in the renal vessels but has also been observed in the distal tubules and cortical collecting ducts, whereas the ET_B_ receptor is primarily found in vasa recta and inner medullary collecting duct (IMCD) but has also been found in proximal tubules and glomerular cells (405). This differential distribution of the ET receptors in the vascular and tubule system might suggest that the receptors can have differential effects.

ET-1 can promote cell proliferation, inflammation, and fibrosis. Increased ET-1 mRNA levels and plasma levels have been observed in 24-h UUO mice (406, 407). In addition, several studies have reported increased expression of *Ednra* mRNA in response to 5-day UUO, whereas the level of the *Ednrb* mRNA expression was not affected by UUO (408–410), suggesting that ET-1 interaction with the ET_A_ receptors might play a role in the progression of interstitial fibrosis in response to UUO. Moreover, treatment with an ET_A_ and ET_B_ receptor antagonist, bosentan, attenuated UUO-induced fibrosis (407) as well as renal dysfunction associated with UUO (86, 406). Cotreatment with bosentan and the AT1 antagonist valsartan had additive protective effects against UUO-induced fibrosis (407). Taken together, these data suggest that inhibition of ET-1 signaling has favorable effects on renal hemodynamics as well as anti-inflammatory and antifibrotic effects in UUO models (FIGURE 9). Interestingly, in a recent study, Neder and colleagues (409) showed increased expression of *Edn1* mRNA in the 5-day UUO kidney in endothelial and tubular cells. They also showed that the increased expression of *Ednra* in the UUO mice mainly occurred in the stromal cell compartment, including renal interstitial fibroblasts. Nevertheless, in a genetic mouse model with constitutive deletion of both endothelin receptors from the stromal cell compartment, there was no change in the UUO-induced expression of proinflammatory and profibrotic markers (409), suggesting that endothelin signaling in stromal cells has a minor effect on renal fibrosis.

#### 8.5.6. Endothelin system as therapeutic target in CKD.

Previous studies have demonstrated increased ET-1 plasma levels in CKD patients, which were associated with kidney function and albuminuria (404). Several clinical trials have tested the therapeutic potential of ET_A_ antagonists in CKD, and early-phase clinical trials suggest that ET_A_ antagonists might be beneficial as nephroprotective and antiproteinuric drugs for diabetes, hypertensive nephropathy, and CKD (411–414). A phase 3 study (ASCEND) assessed the effect of the ET_A_ antagonist avosentan on renal disease progression or death in 1,392 patients with stage 3–4 CKD. At the dosages used, avosentan induced a marked decrease in proteinuria after a median follow-up of 4 mo, but the trial was prematurely terminated because of adverse cardiovascular events in the avosentan groups (238). It was suggested that the use of doses of 25 and 50 mg avosentan may activate the ET_B_ receptor to promote additional fluid retention (415). Taken together, it has been shown that inhibition of ET_A_ receptors can mitigate renal injury and fibrosis at multiple levels. However, the major drawback of endothelin receptor antagonists is the adverse effects of fluid retention, which can potentially be minimized by careful patient selection, diuretic administration, and drug dosing.

### 8.6. Signaling Networks

To add to the complexity, all of the above-described signaling pathways interact, and work in concert, in both an autocrine and a paracrine fashion. In the healthy kidney, this can convey renoprotection (416); however, in the setting of renal fibrosis, this interaction creates an autoinductive feedforward loop resulting in perpetual activation of the profibrotic machinery (326, 417). In FIGURE 10 we provide a simplified overview of how the DAMP, prostanoid, purinergic, and TGFβ signaling pathways can interact and influence the key cellular players in fibrosis. Much more research is needed to elucidate the complex signaling networks, but doing so will hopefully unveil means to attenuate the self-sustaining character of renal fibrosis.

### 8.7. MicroRNA in Obstructive Nephropathy

MicroRNAs (miRNAs) are short noncoding RNAs that interact with 3′-untranslated regions of mRNAs (418). miRNAs modulate gene expression by regulating mRNA translation as a posttranscriptional regulator and provide novel insights into the regulation of protein expression and function (419, 420). For example, miR-32 and miR-137 target AQP2, providing a novel mechanism for the regulation of AQP2 abundance (420, 421). Moreover, aldosterone decreased miR-34c-5p levels in mouse cortical collecting duct cells, and a consequent increase in calcium/calmodulin-dependent protein kinase (CaMK)-IIβ expression could play a role in aldosterone-induced fibrosis (419). miRNAs and miRNA-expressing exosomes produced by cells are involved in the development of renal fibrosis (422–425). For instance, several miRNAs, such as miR-34a (426), miR-21 (424), miR-150-5p (427), miR-216a (428), miR-153-3p (429), miR-215, miR-199a-5p, and miR-199a-3p, have been demonstrated to be upregulated and/or be involved in the development of renal fibrosis in the UUO model (430) (FIGURE 8). miR-21-expressing exosomes promote renal fibrosis after 7-day UUO and target phosphatase and tensin homolog (PTEN) (424). Exosomal miR-150-5p and miR-216a derived from the tubular cells are also involved in renal fibrosis (427, 428). The results indicate that cell-cell communication via exosomes derived from tubular cells could play a role in the pathogenesis of renal fibrosis after UUO, and miRNAs expressed in the exosome are novel therapeutic targets against the progression to CKD.

In contrast, miR-29, which has antifibrotic activity through direct inhibition of YY1 and TGFβ3, partly suppressed renal fibrosis in mice with 14-day UUO when injected in exosome-encapsulated form (431, 432). RNA-seq study in mice subjected to UUO for 5 and 10 days revealed that miR-9-5p mitigated renal fibrosis by preventing downregulation of genes associated with critical metabolic pathways in obstructive nephropathy, including mitochondrial function, oxidative phosphorylation, fatty acid oxidation, and glycolysis (433). Overexpression of the miR-200b/c family attenuated renal fibrosis by controlling EMT via targeting fascin-1 and CD44 (422). Moreover, overexpression of miR-27b-3p in UUO inhibited renal fibrosis via downregulation of signal transducers and activators of transcription 1 (STAT1) expression (434). Thus, miRNAs could be novel therapeutic targets against CKD progression.

### 8.8. Mesenchymal Stem Cell-Derived Extracellular Vesicles in Obstructive Nephropathy

Intrarenal delivery of autologous mesenchymal stem cell (MSC)-derived extracellular vesicles (EVs) has been demonstrated in several studies to ameliorate structural and functional decline and to attenuate renal inflammation, manifested as decreased levels of proinflammatory cytokines, including TNFα, IL-6, and IL-1β (435). Intravenous administration of MSC-derived EVs mitigated tubular injury and fibrosis after 14-day UUO (436). Importantly, miRNAs transferred through EVs are capable of modulating fibrosis and EMT. Consistent with these findings, in vitro experiments using tubular cells treated with TGFβ showed that coincubation with kidney-resident MSC-derived EVs reversed EMT and TGFβ-induced morphological changes. This mechanism was also confirmed in another study on TGFβ-treated endothelial cells, in which MSC-derived EVs ameliorated EMT and improved cell proliferation 7 days after UUO (437). In light of these few studies, MSC-derived EVs may have a role as antifibrotic and renoprotective treatment. Moreover, genome-engineered mesenchymal stem cells secreting a specific protein expressed in the EV could be exploited for effective renoprotective treatment against kidney injury (438). In a recent review, it was concluded that the concept of EV-based treatment for CKD remains promising, but more research is needed regarding standardization of EV protocols, improving study quality, determining the optimal delivery and dosage of EVs, and, most importantly, understanding the global biological mechanisms of the observed protective effects (439).

## 9. CONCLUSIONS

Ureteral obstruction, and its impact on the kidney, has fascinated scientists for decades. To this day, experimental UO remains an important tool in nephrology research. As comprehensively described above, the models of UO have led to the elucidation of numerous aspects of renal (patho)physiology; however, there are many outstanding questions.

One of the challenges to comprehensively understanding the pathophysiological effects of UO has been the lack of studies on molecular changes and changes in renal functions in the same individual. Recently, Wagner and colleagues (440) developed a novel approach to quantify the effects of chronic 5-wk UUO on glomerular processes using two-photon microscopy. This technological improvement may represent a paradigm shift in the possibility of investigating physiological and pathophysiological processes, because it allows in vivo quantification of glomerular capillary blood flow, vasoconstriction, and vasodilation responses to drugs, permeability, and inflammation in the renal tissue.

The pathological processes following UO involve a wide variety of cells; however, proximal tubule cells, (myo)fibroblasts, and macrophages have garnered the most attention from the scientific community, especially in relation to renal fibrosis. In several studies, it has been clearly shown that cells from the distal nephron also contribute to the development of fibrosis. We expect that combining the UO models with advanced genomic analysis techniques, such as scRNA-seq, snATAC-seq, or spatial transcriptomics, will greatly improve our understanding of the pathogenesis of renal fibrosis and will aid in the identification of novel therapeutic targets. Unfortunately, all these genomic techniques share a caveat, that is, a lack of consensus regarding cell type markers, a problem plaguing lineage-tracing studies as well. This is a major issue that the scientific community needs to tackle. Fortunately, reference atlases are continuously growing and annotation protocols are improving.

Besides changes in the cellular composition of the kidney following UO (FIGURE 7), there are also clear changes in the activity of metabolic pathways, such as reduced operation of gluconeogenesis and fatty acid oxidation (FIGURE 5). This aspect is relatively unexplored, especially with regard to distinct metabolic changes at the cellular level as well as their functional consequences. Moving forward, single-cell proteomic and metabolomic analyses should provide essential spatially resolved biochemical information. Together with an improved understanding of key cellular players, we believe that these mass spectrometry studies will increase the catalog of clinically relevant targets and biomarkers.

Another area that needs more attention is the identification and validation of proper biomarkers of CKD-associated renal fibrosis, especially since the extent of fibrosis correlates with, and predicts, renal function loss (441, 442). To date, renal biopsy remains the most widely used method to assess the extent of fibrosis, yet needle-based biopsies are associated with several limitations, one of the most important being that they are not suitable for long-term monitoring of disease progression or therapy responses. This has a big impact on the clinical management of CKD as well as on drug development. In this light, models of UO might aid in the search for novel fibrosis biomarkers, for instance, related to ECM turnover (443, 444). Moreover, we believe that the addition of the “omics” techniques will expedite the identification of novel liquid biomarkers; however, we expect that noninvasive imaging methods, such as elastin imaging (445), may be an important part of the solution and therefore deserve more attention.

Finally, it is of paramount importance to develop new therapies for the treatment of CKD. The most commonly used therapies target conventional causes of CKD, such as hypertension and diabetes, but do not act directly on the kidney. This partly changed with the introduction of SGLT2 inhibitors as CKD therapeutics (FIGURE 11 and TABLE 1). This class of drugs includes dapagliflozin, which was recently approved by the FDA to reduce the risk of decline in kidney function, kidney failure, death caused by cardiovascular diseases, and hospitalization for heart failure in adults with CKD (446). Despite these exciting advances, there remains an urgent need for therapies that can attenuate renal fibrosis, which plays a key role in CKD progression.

One of the main hurdles in developing efficacious antifibrotic therapies is that the majority of profibrotic factors, such as TGFβ, also play key roles in normal physiological processes, thereby markedly increasing the risk for adverse effects following systemic drug therapy. This issue could be mitigated by developing therapeutics that solely act on the diseased organ, for instance, by conjugating drugs to delivery systems that are directed to transporters exclusively expressed in the kidney. Advances have been made in this field (447), but, unfortunately, targeted therapeutics are still not available in clinical practice. With regard to obstructive nephropathy, an aspect that requires attention is the increased risk of CKD and ESRD in some patients after obstruction release. Clinical management of the obstruction and underlying cause might not be sufficient to prevent the deterioration of renal function, and new treatment modalities should incorporate means to reduce the risk of renal failure. Since all experimental models have limitations, future research initiatives with new models should aim to parallel clinical disorders more closely.

To achieve these goals, the various preclinical models of UO will remain an indispensable and relevant tool, with a proven track record dating back to the 1800s.

## GRANTS

This work was supported by grants from the Novo Nordisk foundation (NNF19OC0054481) to R.N., Lundbeck foundation (R368-2021-726) to H.A.M.M., Danish Council for Independent Research (DFF-7016-00305B) to J.F., and the National Research Foundation of Korea (NRF) grant funded by the Korea Government (MIST) (2021R1A5A2021614) and the Korea Health Technology R&D Project through the Korea Health Industry Development Institute (KHIDI), funded by the Ministry of Health & Welfare, Korea (HI15C0001) to T.-H.K.

## DISCLOSURES

No conflicts of interest, financial or otherwise, are declared by the authors.

## AUTHOR CONTRIBUTIONS

R.N., H.A.M.M., J.F. and T.-H.K. prepared figures; drafted manuscript; edited and revised manuscript; and approved final version of manuscript.
